# The Tarsometatarsus of the Ostrich *Struthio camelus*: Anatomy, Bone Densities, and Structural Mechanics

**DOI:** 10.1371/journal.pone.0149708

**Published:** 2016-03-25

**Authors:** Meagan M. Gilbert, Eric Snively, John Cotton

**Affiliations:** 1 Department of Geological Sciences, University of Saskatchewan, Saskatoon, SK, Canada; 2 Department of Biology, University of Wisconsin-La Crosse, La Crosse, WI, United States of America; 3 Mechanical Engineering and Biomedical Engineering, Russ College of Engineering and Technology, Ohio University, Athens, OH, United States of America; College of the Holy Cross, UNITED STATES

## Abstract

**Background:**

The ostrich *Struthio camelus* reaches the highest speeds of any extant biped, and has been an extraordinary subject for studies of soft-tissue anatomy and dynamics of locomotion. An elongate tarsometatarsus in adult ostriches contributes to their speed. The internal osteology of the tarsometatarsus, and its mechanical response to forces of running, are potentially revealing about ostrich foot function.

**Methods/Principal Findings:**

Computed tomography (CT) reveals anatomy and bone densities in tarsometatarsi of an adult and a young juvenile ostrich. A finite element (FE) model for the adult was constructed with properties of compact and cancellous bone where these respective tissues predominate in the original specimen. The model was subjected to a quasi-static analysis under the midstance ground reaction and muscular forces of a fast run. *Anatomy–*Metatarsals are divided proximally and distally and unify around a single internal cavity in most adult tarsometatarsus shafts, but the juvenile retains an internal three-part division of metatarsals throughout the element. The juvenile has a sparsely ossified hypotarsus for insertion of the m. fibularis longus, as part of a proximally separate third metatarsal. Bone is denser in all regions of the adult tarsometatarsus, with cancellous bone concentrated at proximal and distal articulations, and highly dense compact bone throughout the shaft. *Biomechanics–*FE simulations show stress and strain are much greater at midshaft than at force applications, suggesting that shaft bending is the most important stressor of the tarsometatarsus. Contraction of digital flexors, inducing a posterior force at the TMT distal condyles, likely reduces buildup of tensile stresses in the bone by inducing compression at these locations, and counteracts bending loads. Safety factors are high for von Mises stress, consistent with faster running speeds known for ostriches.

**Conclusions/Significance:**

High safety factors suggest that bone densities and anatomy of the ostrich tarsometatarsus confer strength for selectively critical activities, such as fleeing and kicking predators. Anatomical results and FE modeling of the ostrich tarsometatarsus are a useful baseline for testing the structure’s capabilities and constraints for locomotion, through ontogeny and the full step cycle. With this foundation, future analyses can incorporate behaviorally realistic strain rates and distal joint forces, experimental validation, and proximal elements of the ostrich hind limb.

## Introduction

The ostrich (*Struthio camelus*) is the largest and fastest extant ratite, with great capacity for long distance locomotion [1,2,3,4]. The morphology of the ostrich hindlimb has been the subject of numerous studies [1,5,6,2,4,3,7]. High-powered muscle output is channeled through a multi-jointed system of interconnected bi- and tri-articular muscles [6,3] (Table 1). The distal tibiotarsus and tarsometatarsus are elongated and lightened to provide more efficient locomotor capability, with the muscle forces transmitted to the pes via long tendons [8,9,7]. Distal limb length is further extended by elevating the metatarsophalangeal joint above ground [7]. This articulation enhances elastic storage shock absorption during locomotion [2,4]. Ostriches have further reduced distal limb mass by eliminating the second digit and associated musculature, and are thus the only known didactyl birds [3].

**Table 1:** Material properties assigned to FE model of the ostrich tarsometatarsus, and yield and ultimate values for comparison with FEA results. *E* = Young’s modulus (stress/strain), *G* = shear modulus, *ᴠ* = Poisson’s ratio, *σ*_*yield*_ and *σ*_*ult*_ = yield and ultimate stresses, *ε*_*ult*_ and *ε*_*ult*_ = yield and ultimate strains, *ε*_ult_* = strain up to which cancellous bone retains some load-carrying ability. Moduli are in GigaPascals (GPa), and *σ*_*yield*_ and *σ*_*ult*_ in MegaPascals (MPa). Yield and ultimate stresses are reported along the z (long) axis because experimental test samples are typically oriented along this axis, in uniaxial tension or compression tests, and in bending tests which cause compression and tension along different sides of the long axis. The rationale behind this materials testing practice is that most in-vivo loads of limb elements are assumed to be primarily oriented longitudinally. Sources: R = Reed and Brown (2001), M = Martin et al. (1998), K = Keaveny et al. (2004), L = Linde et al. (1992).

Functional group/muscle	Input F for FEA	Reaction F from FEA	Assumed quiescent	FEA constraint
Ankle extension				
M. fibularis longus	✓			
M. flexor hallicus longus	✓			
M. gastrocnemius (all heads)	✓			
Phalangeal flexion			one analysis	
M. flexor perforans et perforatus digiti III	✓			
M. flexor perforatus digiti III	✓			
M. flexor perforatus digiti IV	✓			
M. flexor digitorum longus	✓			
Phalangeal extension			one analysis	
M. extensor digitorum longus	✓			
M. extensor proprius digiti III	✓			
M. extensor brevis digiti III	✓			
M. extensor brevis digiti IV	✓			
Ligamentous function				
M. fibularis brevis				✓
Hip flexion		✓		
Hip extension		✓		
Hip adduction		✓		
Hip abduction		✓		
Knee flexion		✓		
Knee extension		✓		
Internal rotation		✓		
Ankle flexion			✓	

The internal anatomy of the ostrich tarsometatarsus, and its distributions of compact and cancellous bone, have yet to be illustrated in detail or assessed in relation to their structural response during locomotion. We describe the external and internal anatomy of the ostrich tarsometatarsus based on specimen observations, reviews of the literature, and computed tomographic (CT) scans. Pertinent to ostrich biomechanics, we illustrate mineral densities of adult and juvenile tarsometatarsi, and present a finite element (FE) model of an adult’s tarsometatarsus, which is useful for simulating its response to external loads. Our goals are 1) to present osseous anatomy and densities of the tarsometatarsus; 2) to relate anatomy and densities to stresses and strains under a sample loading regime for the adult, and 3) to identify refinements for locomotor finite element analysis (FEA) of individual limb bones. Our goal is not to claim definitive anatomical/functional correlations, when the accuracy of the most thorough models of ostrich locomotion is unknown [10], even when carefully compared to *in vivo* experimental data. Instead, the anatomical data and FE model will be useful for future analyses of structural response through the step cycle (during both running and turning: [10]). Forces for such studies will be derived from experimental data and motion simulation through methods of multibody kinematics and dynamics [5,11,2,4,12,13]. The current study is a first and necessarily simplified stage of correlating anatomy with FEA, which will ultimately combine simultaneous multibody dynamics (MBD) and FEA, as Snively, Kumbhar et al. (2013) [14] have applied to chewing pigs. The current ostrich loading regime is a “snapshot” (single time-increment) from manual MBD calculations, which are presented here fully for replication and as a guide to coding in programs such as MATLAB and Mathematica. This approach is a transparent complement to off-the-shelf MBD programs, including MSC Adams and the open-source GaitSym and OpenSim, which researchers can use as efficient black-box solutions for calculating muscle and reaction forces in many poses.

### Interpretation of CT bone densities in juvenile and adult *Struthio camelus*

We compare densities of juvenile and adult ostrich tarsometatarsi to assess changes in ontogenetic and locomotor adaptation. Ostriches grow quickly, with males growing from 1 to 100 kg within a year as their tarsometatarsi lengthen approximately 8-fold [15]. Their limb bones experience shifts in histology and presumably in density, due to conditions necessary for fast growth in juveniles (including calcified cartilage interdigitated with long marrow tubes for bone deposition: [16,17]) to denser, stiffer elements essential for rapid locomotion in adults. CT scans enable us to assess densities of mineralized tissues. These densities are directly proportional to stiffness ([18,19,20], and thus resistance to deformation from locomotor forces.

Densities are often expressed as Hounsfield units (HU) [19], indexed from x-ray attenuation values, which enable comparison of tissue density to that of water. A Hounsfield value of 0 is that of water, corresponding to a density of 1 g/cm^3^. For air the HU value is -1,000, and bone ranges from 700 HU for dense cancellous to 3000 HU for highly dense compact bone.

We scanned juvenile and adult tarsometatarsi on medical scanners (see Materials and Methods), and further scanned proximal and distal ends of the juvenile specimen on a micro-CT scanner. Most medical-resolution scanners differentiate a 12-bit range of densities, 4,096 Hounsfield units wide, and the micro-CT has 16-bit resolution of 65,536 density levels (not indexed as traditional HU). Because the juvenile TMT had a narrow range of density, the micro-CT scan revealed gradations of its density not possible with the medical scanner. To visualize these gradations and highlight density variation, we use full-color palettes ([21,22]) instead of solely using a greyscale range. To best visualize densities, each color scale is centered on an average HU level with a range around it. HU color scales differ for the adult and juvenile because the average density is lower and range of densities smaller in the juvenile.

### Finite element analyses: properties, loadings and constraints

Bone density and stiffness are testably correlated with stresses and strains from locomotor force [23], using finite element analysis [23,24,25]. Table 2 lists stiffnesses of and other properties of bone used in this study. FEA calculates mechanical response of structures modeled as a mesh of small elements, connected at nodes and assigned stiffness and other properties of the original material. The model is constrained to prevent rigid body motion, subjected to external forces, and the resulting displacement of nodes enables calculation of stresses (internal force/area), strains (change in dimension/initial dimension), and reaction forces.

**Table 2:** Muscle forces applied to the ostrich tarsometatarsus (TMT), and subsumed into finite element reaction forces at the mesotarsal joint. Forces for ankle extensors are calculated as necessary to counteract the ground reaction moment, and in one analysis digital flexor and extensor forces are applied to stabilize the TMT-phalangeal joints. Effects of muscles acting on proximal limb elements emerge from FEA as joint reaction forces at the proximal surface of the TMT.

Bone:	Axes	*E* GPa	*G* GPa	*ᴠ*	*σ*_*yield*_ MPa bend. compr. tens.	*σ*_*ult*_ MPa bend. compr. tens.	*ε*_*yield*_ % bend. compr. tens.	*ε*_*ult*_ % bend. compr.tens
Compact	x	9.04	-	0.3				
	y	9.04	-					
long axis	z	15.86	-		126.54^R^ 182–196^M^ 115–141^M^	154.6^R^ 195–237^M^ 133–156^M^	0.68^R^ 1.3^K^ 1^K^	1.03^R^ 2^K^ 3^K^
	xy	-	3.65					
	xz	-	5.10					
	yz	-	5.10					
Cancellous	iso.	0.64		0.29	20^K^-30^K^		0.6^K^-0.9^K^	1.55^K^-2^L^

We constructed a finite element model of the adult *Struthio* tarsometatarsus in a quasi-static, mid-stance pose in which ground reaction force of running would be vertical and at its greatest magnitude. The FE model was constrained at the ankle joint by ligaments and contact with the tibiotarsus. Components of the ground reaction force *F*_*GRF*_ on the distal end of the tarsometatarsus and muscle force magnitudes and directions necessary to counteract *F*_*GRF*_, were calculated in the “ground” coordinate system, with the vertical z-axis in line with the ground reaction force (see Materials and Methods for details). Because forces were applied to the FE model in its “anatomical” coordinate system (with z along the TMT proximodistal axis), we shifted force directions using coordinate frame rotation common in robotics [26].

Forces from other muscles and ligaments (including the reduced M. fibularis brevis) not necessary to extend the tarsometatarsus were accounted for (Table 2) through the applied ground force, derived from reaction forces at finite element constraints, or applied as stays that would counteract bending. Proximal muscles (not inserting on the TMT) held the femur and tibia stiff to hold the body over the ankle joint, contributing to the ankle’s simulated instantaneous constraint (and resulting reaction force) in this simulated quasi-static pose. The phalangeal flexors and extensors were considered as momentary stabilizers of the TMT-phalangeal joints, and thus contributed to the applied loading at these joints both subsumed into the vertical joint reaction force and with additional applied horizontal force in a sensitivity analysis.

We undertook several such sensitivity analyses, to examine the effects of model resolution, material properties, and loading regimes including varying muscle forces.

1. We ran convergence analyses to test how closely our initial model approached the peak stress results of high-resolution models, and thus to test its suitability for testing the effects of many different loads and material properties.
2. In addition to force from the phalanges supporting the tarsometatarsus in the vertical direction, in a separate analysis we applied forces from phalangeal flexors and extensors to simulate the effects of stabilizing loads from these muscles.
3. Unusually, our dissections of a young ostrich showed the M. gastrocnemius tendon inserting partly on a structure called the hypotarsus, along with M. fibularis longus that normally inserts there. To test the effects of both possibilities on TMT stress and strain, we ran analyses with both insertion configurations.
4. As a further test of varying biological parameters, we tested the effects of uniform material properties versus our initial model with both compact and cancellous bone.

### Interpreting FEA results

We apply finite element analysis to discover how the adult tarsometatarsus would respond to the applied loads. Stress (*σ*: internal force/area) and strain (*ε*: proportional deformation) results from FEA enable us to visualize distribution and magnitude of these quantities, and to estimate how close a structure is to breaking (strength, or ultimate stress or strain: *σ*_*ult*_ or *ε*_*ult*_) or permanent deformation (yield: *σ*_*yield*_ or *ε*_*yield*_). FEA differentiates between axial (compression or tension) and shear stress and strain (Appendix 1), and calculates principal stresses and strains as eigenvectors, similar to principal components in multivariate statistics. To assess damage in bone, we use von Mises stress and strain, *σ*_*vM*_ and *ε*_*vM*_, which are functions of principal values that correlate well with failure in experimental tests (see Appendix 1 of the current paper; [25]). Under the von Mises criterion, ultimate stress *σ*_*ult*_ of dense compact bone is about 180–200 MegaPascals (MPa; N/m^2^) in compression, 150 MPa in tension, and 80–100 MPa in shear [23]. von Mises ultimate and yield values are consistent for bone across vertebrate taxa, and are therefore reasonable assumptions for ostrich bone.

Dividing the ultimate or yield von Mises stress by an element’s experienced *σ*_*vM*_, for example, gives a safety factor under the given loading regime [21]. For example, under a compressive load, bone experiencing *σ*_*vM*_ of 20 MPa would have a safety factor of about 10 against breaking, if *σ*_*ult*_ is 200 MPa. Tables of stress and strain at sampled points, and color-coded illustrations of these FE results, enable assessment of safety factors throughout the TMT. Full constraints in FEA give artificially high stresses and strains, and reliable interpretations of safety factor are possible at characteristic distances from the constraint. For example, stresses and strains within a cylinder constrained across the entire surface at one end can be safely interpreted only within the part of the cylinder that is separated from the constrained end by a distance greater than the cylinder’s diameter. Constraints applied to smaller surface areas result in higher (artificial) peak stresses, but enable safer interpretation closer to the constraint.

## Results

### Review of tarsometatarsus external osteology

**Fig 1:**
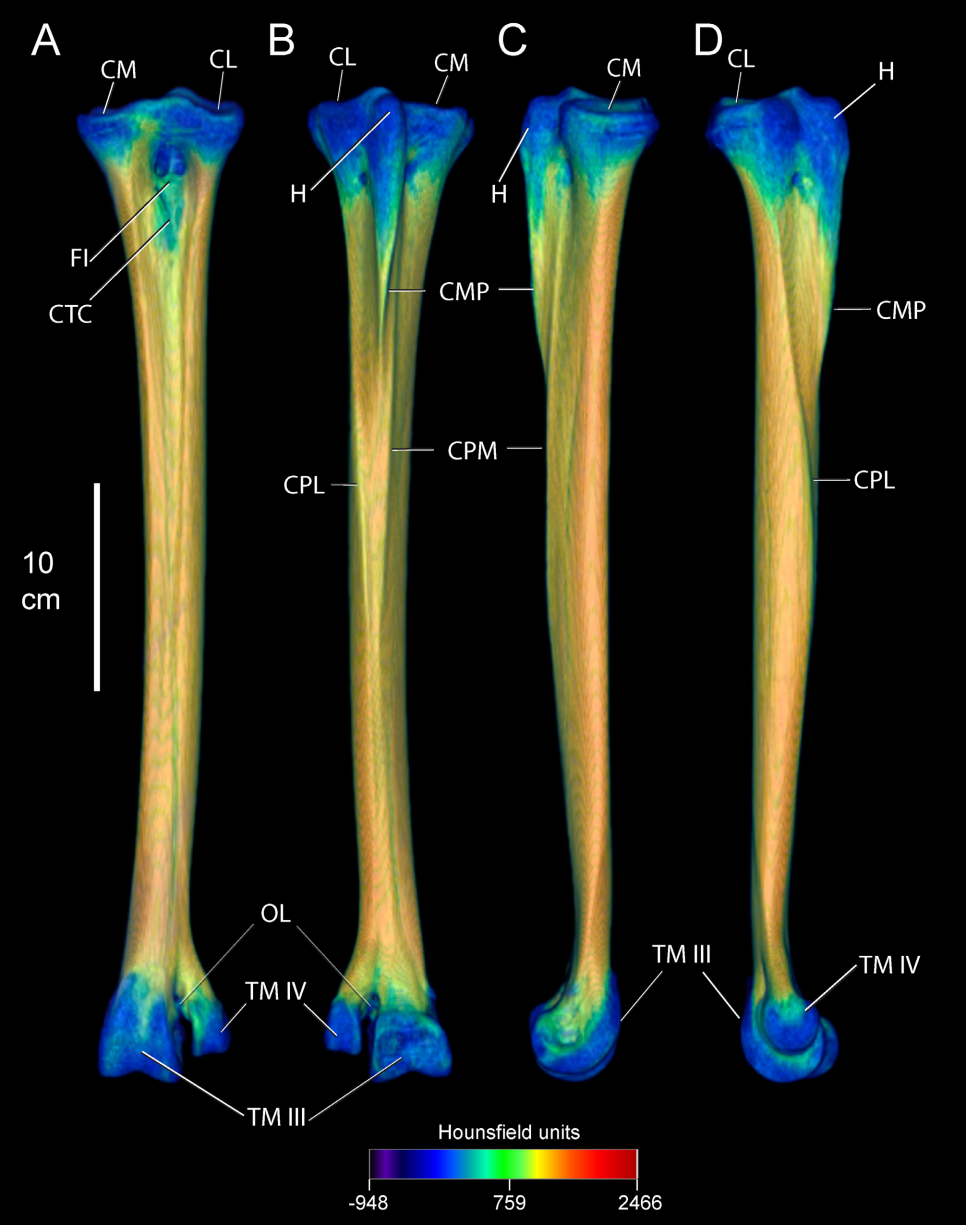
Densities in Hounsfield units (HU) on the external surface of an adult ostrich left tarsometatarsus, reconstructed in anterior (A), posterior (B), medial (C), and lateral (D) views. High-density compact bone occurs throughout the shaft. Low density is present at the articular ends near the mesotarsal and metatarsophalangeal joints. Note that the hypotarsus grades from proximal low density bone to distal high density compact bone. Abbreviations: CL = cotyla lateralis, CM = cotya medialis, FI = fossa infracotylaris, CTC = Crista tibialis cranialis, H = hypotarsus, CMP = Crista medianoplantaris, CPL = Crista plantares lateralis, CPM = Crista plantares medialis, OL = ossified ligament, TM III = trochlea metatarsi III, TM IV = trochlea metatarsi IV.

**Fig 2:**
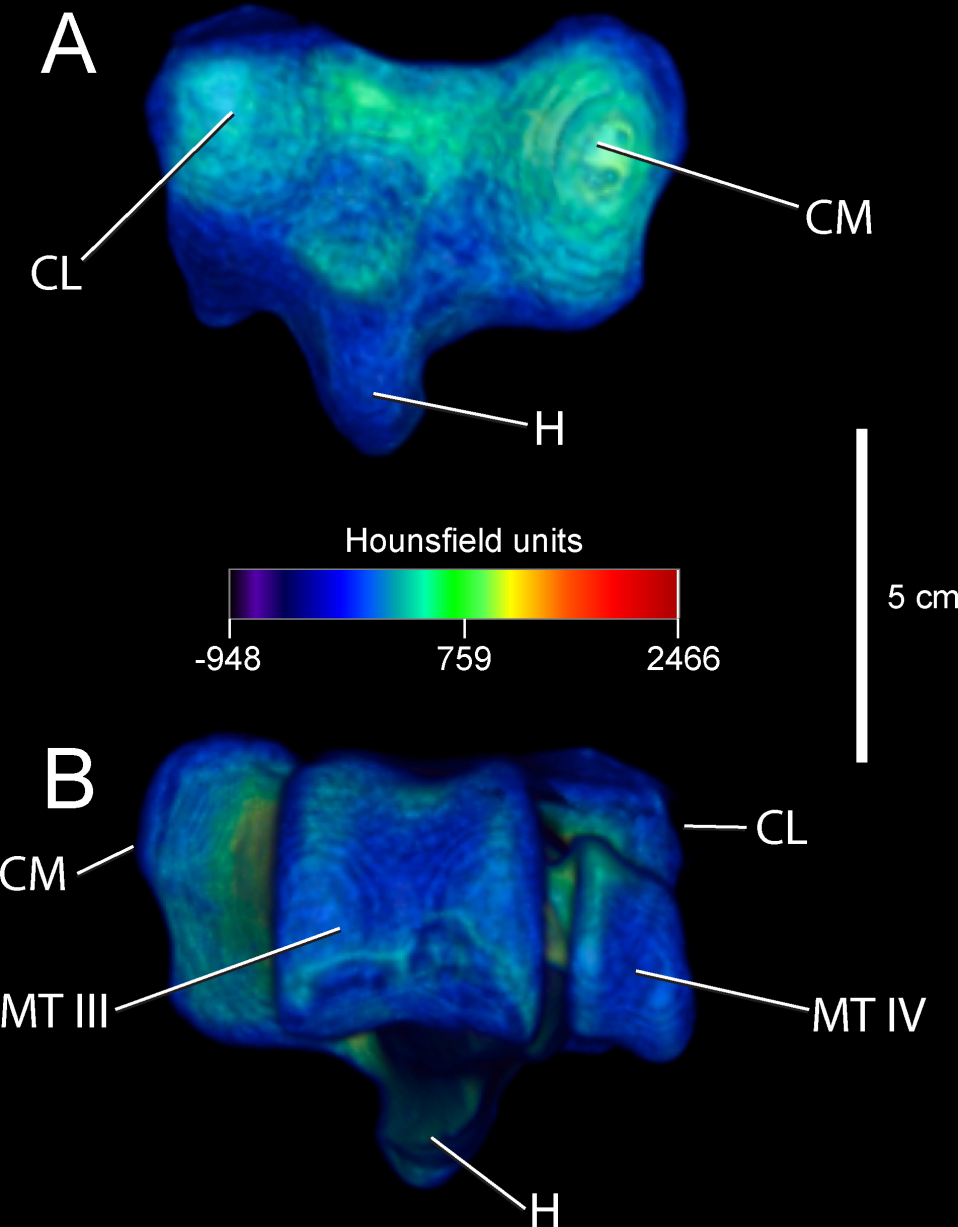
Densities in Hounsfield units (HU on the external surface of an adult ostrich left tarsometatarsus, reconstructed in proximal (A) and distal (B) views. Note bone density at joint surfaces is significantly less dense then that at the shaft. Abbreviations: CL = cotyla lateralis, CM = cotyla medialis, H = hypotarsus, TM III = trochlea metatarsi III, TM IV = trochlea metatarsi IV. Finite element constraints: CM, CL.

**Fig 3:**
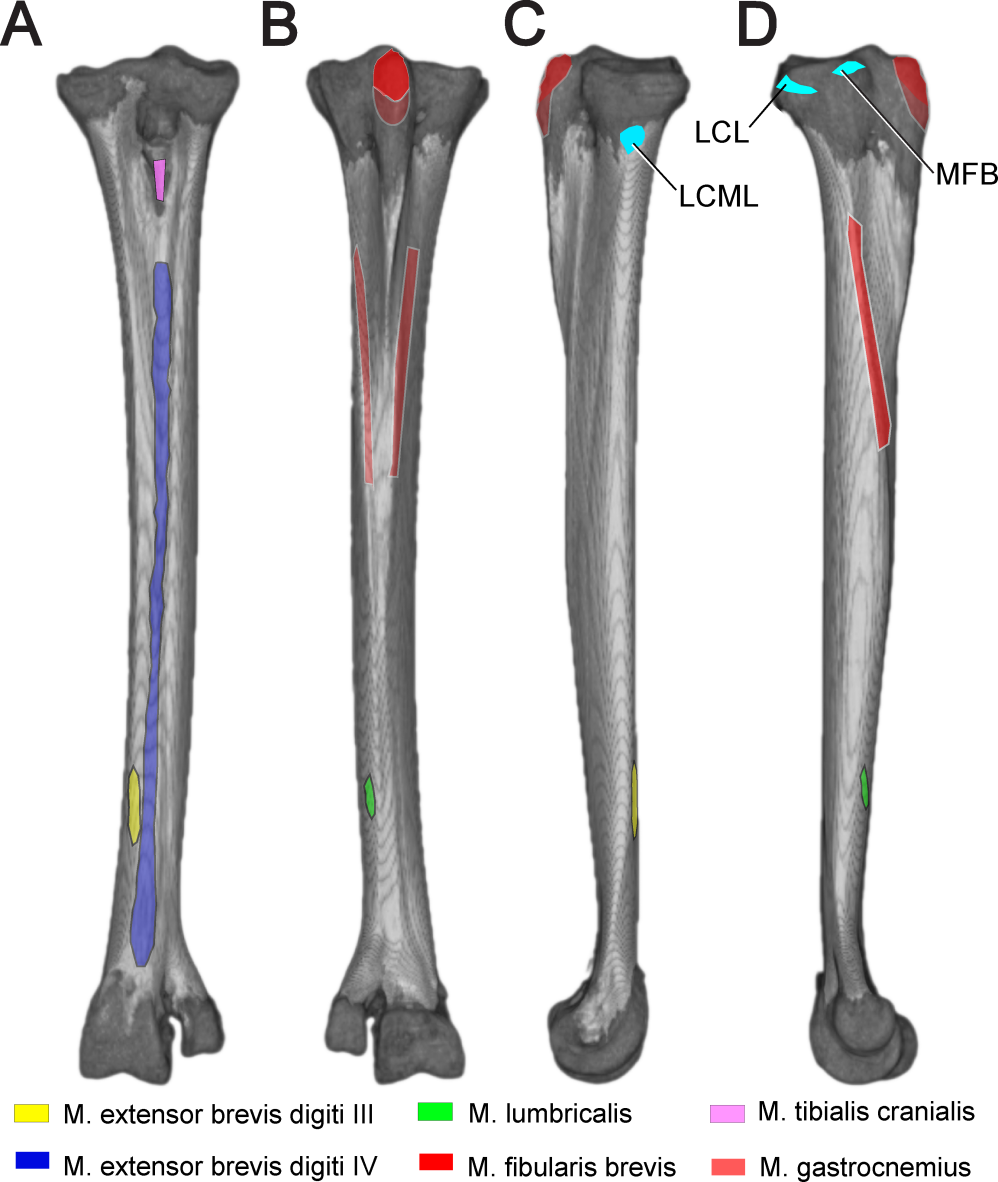
Reconstructed left tarsometatarsus in anterior (A), posterior (B), medial (C), and (D) lateral views, depicting soft tissue attachments. The proximal M. gastrocnemius attachment appeared to be present in the juvenile, but has not been reported in adults. Turquoise coloration indicates ligaments connecting to stabilize the intertarsal joint. Abbreviations: LCM = ligamentum collaterale mediale, LCML = ligamentum collaterale mediale longum, MFB = M. fibularis brevis

Anatomical descriptions are from our dissections and observations, primarily following terminology of Gangl [6] and Smith [9]. Figs 1 and 2 present the external anatomy and bone densities of the adult *Struthio* tarsometatarsus, as rendered from CT scans; labels for Figs 1–3 also associate features with forces and constraints for FEA. As in other birds, the ostrich tarsometatarsus is comprised of fused metatarsal (MT) bones II, III, IV, and the distal tarsals at the mesotarsal joint. Unique among known avian species, MT II does not articulate with phalanges and is externally lost in adults.

Anteriorly, the ostrich tarsometatarsus is broad proximally and slender distally. Proximally, the concave and oval cotyla medialis and cotyla lateralis articulate with the tibiotarsus to form the intertarsal joint. Inferior to the cotyla, the fossa infracotylaris forms a central depression anteroproximally. The crista tibialis cranialis sits within the fossa infracotylaris. Projecting directly posterior to the mesotarsal articular surface is the hypotarsus, where the extensor M. fibularis longus inserts. The hypotarsus grades distally into the Crista medianoplantaris. Ridges of the Cristae plantares lateralis and medials flank the hypotarsus and Crista medianoplantaris, and continue distally. Narrowing and tapering distally, the entire tarsometatarsus shaft forms a cylinder-shaped barrel before expanding and terminating into trochlea metatasi III and IV. MT IV diverges laterally from the main shaft at the foramen vascular distale to form the wedge-shaped trochlea metatarsi IV, attachment for phalanges of digit IV. MT III continues to expand distally past MT IV, becoming trochlea metatarsi III for phalanges of digit III, forming the metatarsophalangeal joint.

### Muscle and ligament attachments

**Fig 4:**
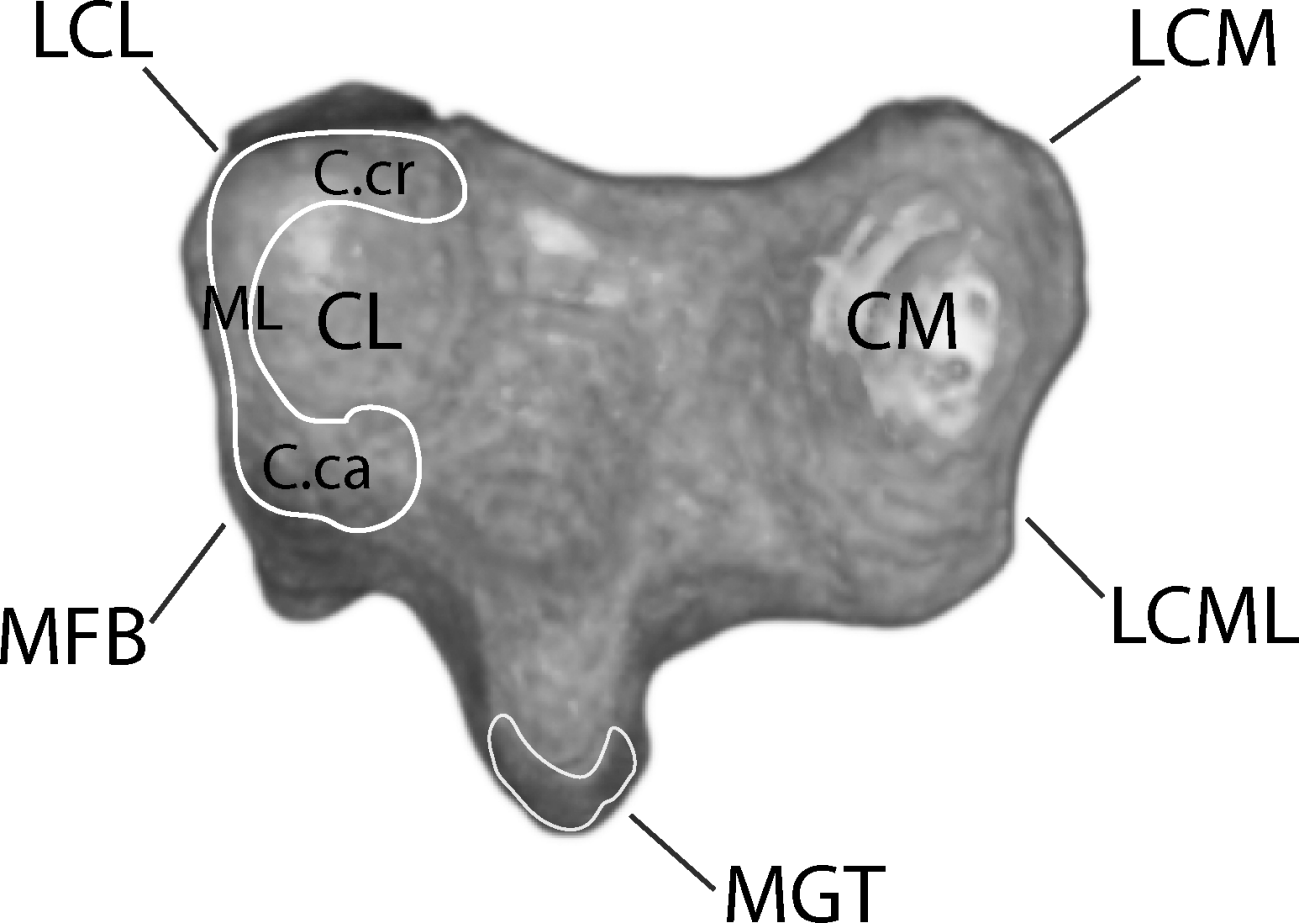
Proximal left tarsometatarsus joint surface showing attachment sites for ligaments and menisci. Note the C-shaped meniscus lateralis at the lateral joint surface. Abbreviations: C.ca = Cornu caudale, C.cr = Cornu craniale, CL = Cotyla lateralis, CM = Cotyla medialis, LCL = lateral collaterale ligament, LCM = ligamentum collaterale mediale, LCML = ligamentum collaterale mediale longum, ML = meniscus lateralis, MFB = M. fibularis brevis, MGT = M. gastrocnemius tendon.

Figs 3 and 4 depict muscle and ligament attachments to the tarsometatarsus, excepting joint capsule entheses. M. gastrocnemius, composed proximally of three separate heads, fuses mid-distal tibiotarsus and inserts posterodistally on the proximal two thirds of the Cristae plantares lateralis and medials of the tarsometatarsus, and in our dissections of the juvenile ostrich appeared to attach to the hypotarsus posterior to an insertion of M. fibularis longus. Proximal to insertion, a fascial sheet connects the lateral edge of the M. gastrocnemius tendon to the tarsometatarsus.

M. tibialis cranialis inserts onto the Crista tibialis cranialis, slipping beneath the Retinaculum extensorium tibiotarsi. Osteological correlates for attachment of the retinaculum, such as Impressiones retinaculi extensori seen in some birds [27], are ambiguous in *Struthio*.

**Fig 5:**
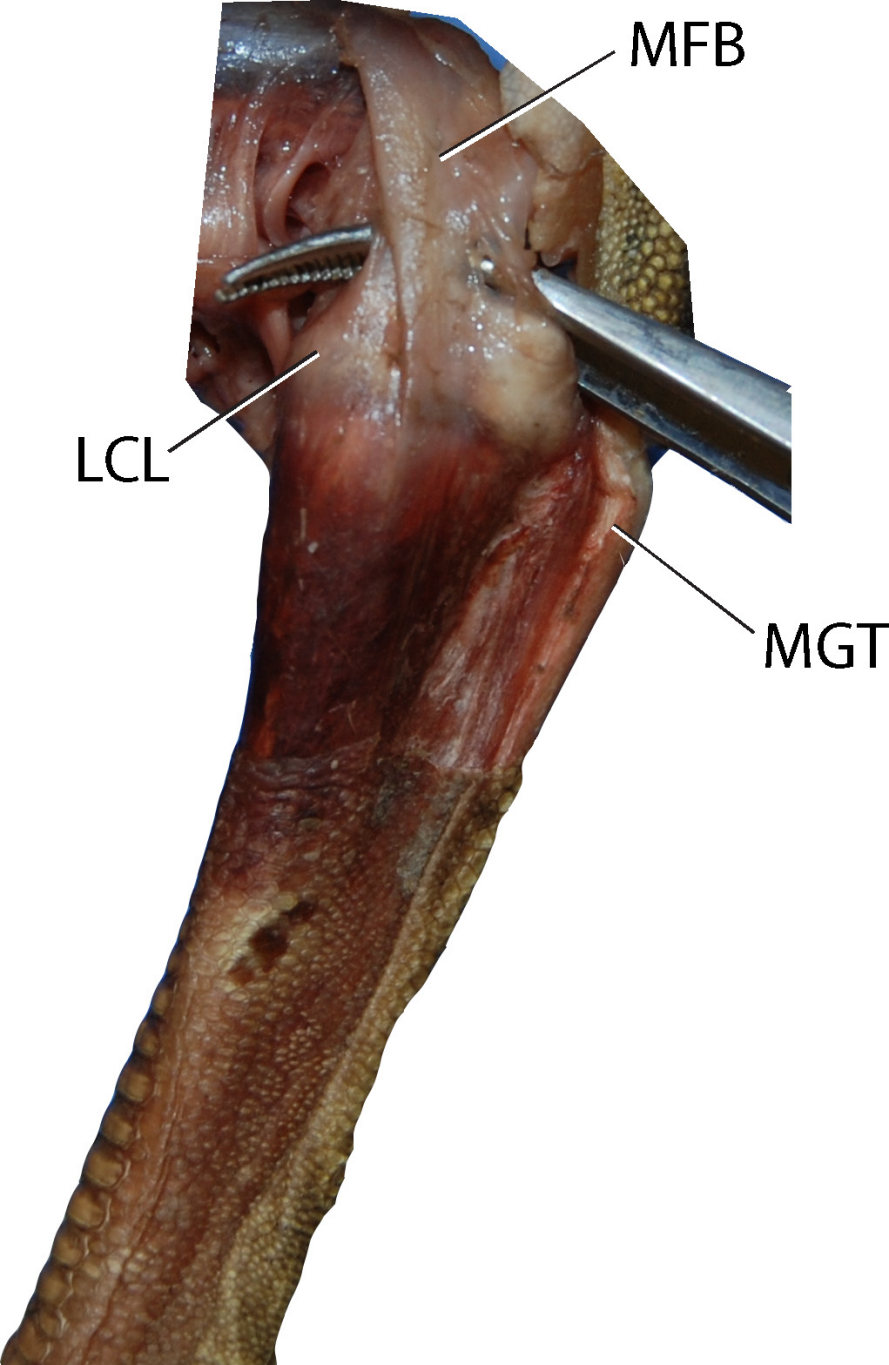
Proximal left tarsometatarsus from a juvenile ostrich. Note the M. fibularis brevis, which is reduced to a tendon in *Struthio*. At full flexion, the MFB crosses the origin of the ligamentum collaterale laterale. Abbreviations: LCL = Ligamentum collaterale laterale, MFB = M. fibularis brevis, MGT = M. gastrocnemius tendon.

M. fibularis brevis originates distolatetal of the fibula on the tibiotarsus. While fully developed in other birds, the M. fibularis brevis is reduced to a tendon functioning as a ligament in *Struthio*. When fully extended the M. fibularis brevis runs transversely and crosses the origin of the Ligamentum collaterale laterale. The muscle inserts on the proxinal plantar surface on the tarsometatarsus. When moving from extension to flexion, the M. fibularis brevis crosses the Ligamentum collaterale laterale at full flexion (Fig 5).

M. fibularis longus originates primarily from the collective tendinofacial sheet associated with the distal femur and proximal tibia, and an aponeurosis from the lateral cnemial crest at the knee. This muscle inserts proximally on the tarsometatarsus, and functions in ankle extension.

M. extensor brevis digiti IV originates dorsally on the distal third of the tarsometatarsus. Passing through the Canalis interosseous tendineus at the distal end of the tarsometatarsus, this muscle inserts medially on the first phalanx of the fourth toe.

M. extensor brevis digiti III originates dorsally on the tarsometatarsus and distomedially of the origin of the M. extensor brevis digiti IV. Attaching dorsal to the joint capsule of the metatarsophalangeal joint of the third toe, this muscle inserts on the dorsal process of the articular cartilage of the proximal articular surface of the first phalanx of the third toe.

M. lumbricalis originates on the dorsal tendon of the M. flexor digitorium longus near the beginning of the distal third of the tarsometatarsus. This muscle divides into two Crura, finally inserting on the Ligamenta plantaria of the metatarsophalangeal joints of the third and fourth toe.

On lateral and medial surfaces, pits occur for collateral ligaments connecting the metatarsals with proximal phalanges. Proximal to the collateral ligament pits, there is a transverse structure of spiculated bone. This appears to be ossified ligament connecting the diverging free shafts of MT III and MT IV.

### CT results: bone densities and internal tarsometatarsus osteology

**Fig 6:**
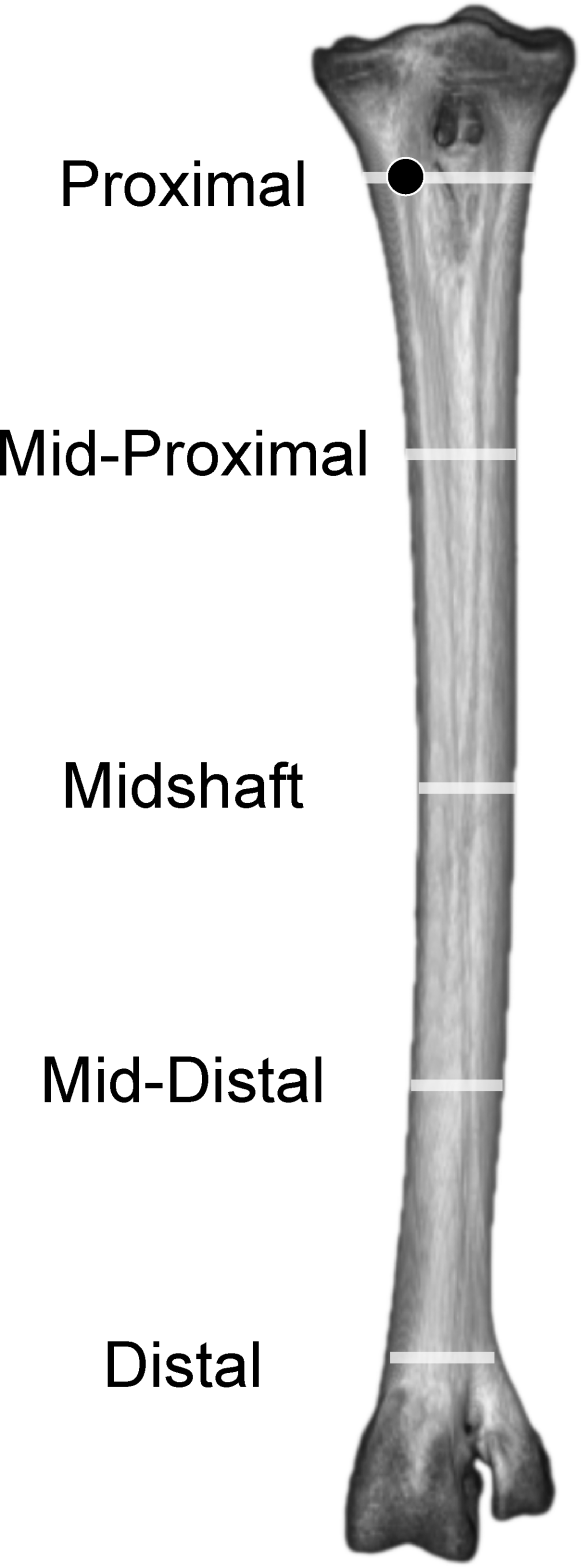
Adult ostrich left tarsometatarsus in anterior view illustrating longitudinal positions of sampled densities, stresses and strains. Results were sampled from anterior, posterior, lateral, and medial surfaces, in the center of the each transect (white lines), except proximally where results were sampled from MT II (black dot) to avoid the Fossa infracotylaris.

**Fig 7:**
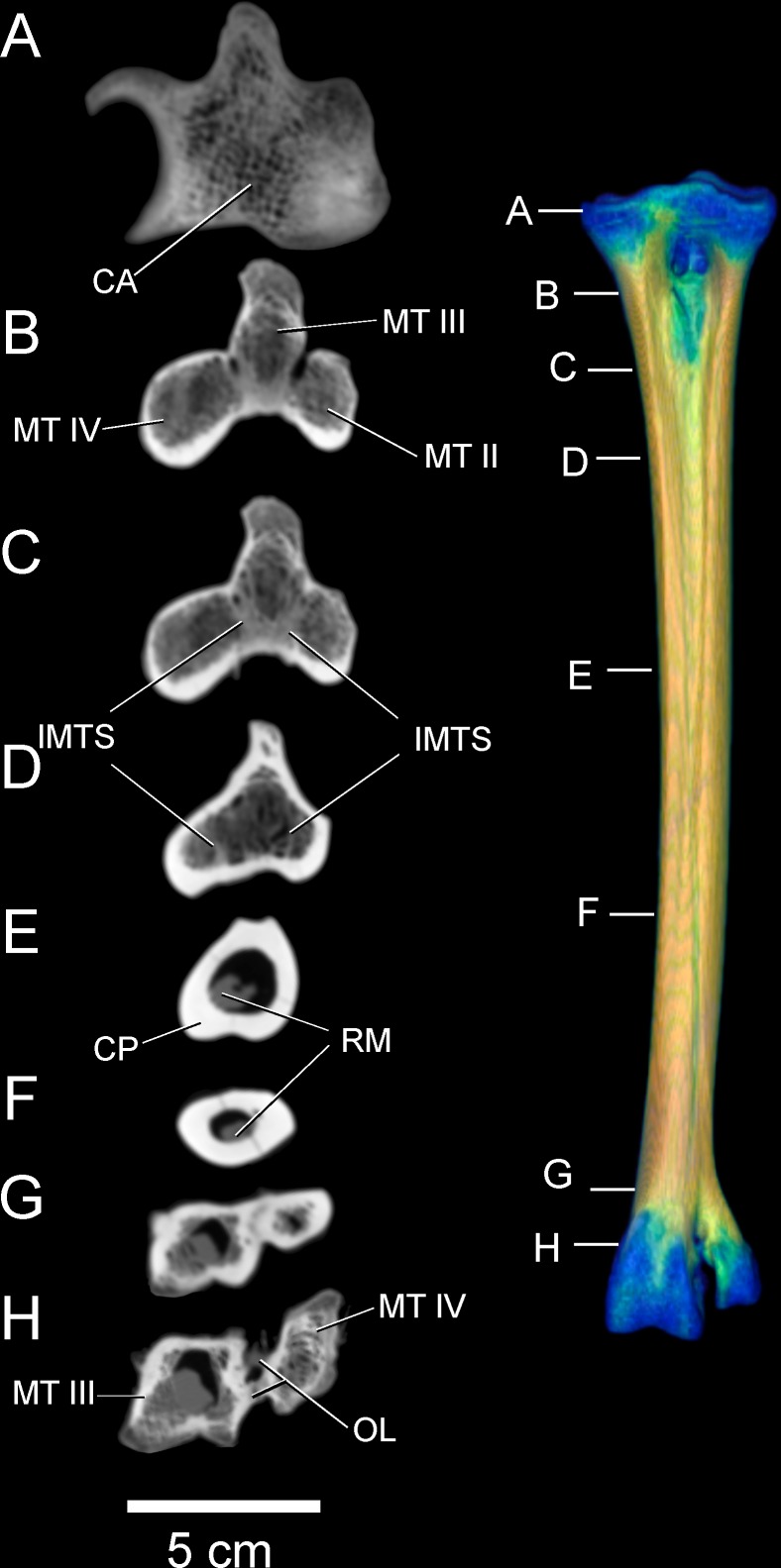
CT cross sections of left adult tarsometatarsus, from proximal (A) to distal (H). Despite ankylosis, distinct metatarsal bones separated by intermetatarsal septa are present in the proximal and distal sections of the bone. The individual metatarsals become fully fused through the center of the shaft, creating a circular, hollow tube composed of compact bone. Abbreviations: CA = cancellous bone, CP = compact bone, IMTS = intermetatarsal septum, MT II = metatarsal II, MT III = metatarsal III, MT IV = metatarsal IV, OL = ossified ligament, RM = residual marrow.

Tables 3 and 4 list relative densities in Hounsfield units on the surface of the adult tarsometatarsus, depicted in Figs 1 and 2, for discrete osteological features and specified locations along shaft (Fig 6). The internal structure of the tarsometatarsus (Fig 7) is complicated by fusion of the distal tarsals and metatarsals II, III, and IV. According to position criterion of homology, MT II is located medially on the shaft, with MT IV lateral, and MT III in the median plane. A high concentration of low-density trabecular bone infuses the distal- and proximal- most articular ends, with little superficial or deep cortical bone present in these regions. Larger struts of bone occur just distal and proximal to the element’s extremities. Trabecular bone gives way to compact bone in the tarsometatarsus shaft. Proximally, three distinct internal cavities are present, separated by partitions of compact bone. In the longitudinally central 50% of the tarsometatarsus shaft in the adult, these partitions are obliterated and there is only one central cavity as MT II, III, and IV fuse completely. MT II narrows distally, tapering to the medial aspect of the tarsometatarsus, where on all other bird species the trochlea metatarsi II would diverge from the main shaft to form an articular surface. Cortical bone thins at the distal end, with trabecular bone increasing in frequency and density. Splitting laterally from the main shaft, MT IV forms trochlea metatarsi IV, comprised nearly exclusively of trabecular bone. MT III continues to flare distally, forming trochlea metatarsi III. Like MT IV, MT III is composed primarily of trabecular bone.

**Table 3:** Hounsfield attenuation units (HU), stresses in the tarsometatarsus’s coordinate system (+x anterior, +y medial, +z proximal), and von Mises stresses *σ*_*vm*_, at anatomical features and positions along the tarsometatarsus shaft. Brick numbers are sampled tetrahedral elements at the specified locations (Fig 7). Abbreviations are as in Figs 1–4; CLP = collateral ligament pit, and ITM IV = the centerpoint of Trochlea metatarsi IV. Positive values indicate tension, and negative indicates compression. Except at constraints (LCML = ligamentum collaterale mediale longum, CL = Cotyla lateralis), the greatest compressive stresses occur along the bone’s long axis (*σ*_*ZZ*_) on the medial and anterior surfaces, and tensile stresses posteriorly and laterally at midshaft and just distal to this (“Mid-distal”). *σ*_*vm*_ are also highest at these positions. Note artificially high stresses where compact and cancellous elements meet in the collateral ligament pit of MT IV, low shear stresses (*σ*_*XY*_, *σ*_*YZ*_, *σ*_*XZ*_) except at constraints, and low stresses at the trochlear applications of the ground reaction force (TM III lateral and medial; ITM IV).

					Stress (MPa)				
Feature:	Brick	HU	*σ*_*XX*_	*σ*_*YY*_	*σ*_*ZZ*_	*σ*_*XY*_	*σ*_*YZ*_	*σ*_*XZ*_	*σ*_*vm*_
CM	132270	244	-2.58	0.95	-0.45	0.14	2.08	0.96	5.03
CL	9221	280	-30.90	-18.60	-27.70	-0.85	1.83	-8.36	18.60
FI	480006	955	1.48	-1.31	-4.30	-0.90	-5.17	0.21	10.40
H	257214	238	0.26	0.83	9.69	-0.15	-1.02	2.32	10.20
CLP III	140472	1278	-0.31	-0.22	-4.03	-0.17	0.86	0.14	4.07
CLP IV	77995	672	1.75	-0.84	-12.40	-0.90	2.54	3.72	15.30
TM III lat	70590	491	0.10	0.48	0.50	0.37	-0.05	-0.02	0.76
TM III med	70666	275	0.03	-0.50	-1.96	-0.23	-0.18	0.17	1.88
ITM IV	376	206	-0.66	0.46	-1.34	0.03	0.86	0.52	2.34
LCL	131524	600	-1.29	-0.59	0.05	-0.59	0.80	0.46	2.23
MFB	8996	257	0.10	0.55	0.20	0.20	0.74	-0.71	1.85
LCML	127255	417	-1.44	-2.56	-18.00	1.62	5.89	-4.29	20.60
Shaft position:					Stress (MPa)				
Anterior	Brick	HU	*σ*_*XX*_	*σ*_*YY*_	*σ*_*ZZ*_	*σ*_*XY*_	*σ*_*YZ*_	*σ*_*XZ*_	*σ*_*vm*_
Proximal	480229	1753	-2.02	-0.88	0.73	2.89	-2.73	-2.62	8.59
Mid-proximal	67642	1642	-1.15	-0.60	-31.10	-0.80	0.30	-0.47	30.30
Midshaft	102208	1590	1.88	-1.71	-33.70	1.16	-0.13	-1.77	34.10
Mid-distal	93774	1767	0.64	-0.53	-31.90	-0.77	-1.47	-3.45	32.60
Distal	2276	1741	0.74	-0.81	-12.60	0.60	-0.02	-0.04	12.70
Posterior									
Proximal	123907	1191	-0.07	-0.22	7.91	0.11	0.25	0.68	8.16
Mid-proximal	238875	1655	1.09	0.89	24.80	-0.51	-1.00	1.87	24.10
Midshaft	100989	1635	-0.54	-0.30	30.80	-0.25	-1.49	0.85	31.40
Mid-distal	94452	1623	0.67	-0.08	25.20	-0.80	1.82	3.48	25.80
Distal	86165	1725	0.53	-1.05	-0.42	-0.02	1.78	-0.36	3.43
Medial									
Proximal	66935	1448	0.19	0.60	-17.30	-0.39	6.82	-1.66	21.40
Mid-proximal	112946	1830	-0.54	-0.70	-25.70	0.29	2.95	-0.05	25.60
Midshaft	22786	1892	-0.32	-0.11	-22.60	0.06	1.62	0.75	22.60
Mid-distal	93732	1848	0.41	0.03	-23.40	1.02	0.60	0.05	23.70
Distal	11522	1822	-0.50	-1.02	-16.20	0.25	-2.51	0.90	16.10
Lateral									
Proximal	123797	1256	-0.41	0.29	-5.78	-0.19	-0.36	-1.91	6.67
Mid-proximal	6052	1721	-0.24	0.63	6.24	-0.19	-0.87	-1.13	6.58
Midshaft	101465	1792	-1.13	0.56	15.40	-0.64	-0.25	1.12	15.90
Mid-distal	93738	1856	2.57	1.27	14.60	0.38	1.14	3.01	13.90
Distal	86766	1830	0.05	0.03	4.02	-0.28	-0.43	0.29	4.11

**Table 4:** Hounsfield attenuation units (HU), strains in the tarsometatarsus’s coordinate system (+x anterior, +y medial, +z proximal), and von Mises strains *ε*_*vm*_, at anatomical features and positions along the tarsometatarsus shaft. Brick numbers are sampled tetrahedral elements at the specified locations (Fig 7). Abbreviations are as in Figs 1–4; CLP = collateral ligament pit, and ITM IV = the centerpoint of Trochlea metatarsi IV. All notable patterns are the same as for stresses, explained in the caption for Table 3.

					Strain (%)				
Feature:	Brick	HU	*ε*_*XX*_	*ε*_*YY*_	*ε*_*ZZ*_	*ε*_*XY*_	*ε*_*YZ*_	*ε*_*XZ*_	*ε*_*vm*_
CM	132270	244	-0.426	0.285	0.004	0.055	0.839	0.387	1.010
CL	9221	280	-2.740	-0.250	-2.080	-0.344	0.737	-3.370	3.740
FI	480006	955	0.033	-0.009	-0.030	-0.032	-0.108	0.019	0.113
H	257214	238	-0.437	-0.320	1.460	-0.062	-0.411	0.935	2.050
CLP III	140472	1278	0.145	0.162	-0.606	-0.069	0.345	0.055	0.820
CLP IV	77995	672	0.060	0.012	-0.083	-0.040	0.042	0.091	0.157
TM III lat	70590	491	-0.029	0.047	0.053	0.149	-0.021	-0.009	0.153
TM III med	70666	275	0.116	0.010	-0.285	-0.092	-0.072	0.068	0.379
ITM IV	376	206	-0.063	0.162	-0.200	0.013	0.345	0.208	0.471
LCL	131524	600	-0.178	-0.035	0.093	-0.239	0.322	0.186	0.449
MFB	8996	257	-0.018	0.072	0.002	0.079	0.297	-0.285	0.372
LCML	127255	417	0.705	0.480	-2.630	0.654	2.370	-1.730	4.150
Shaft position:					Strain (%)				
Anterior	Brick	HU	*ε*_*XX*_	*ε*_*YY*_	*ε*_*ZZ*_	*ε*_*XY*_	*ε*_*YZ*_	*ε*_*XZ*_	*ε*_*vm*_
Proximal	480229	1753	-0.027	0.001	0.016	0.073	-0.049	-0.062	0.100
Mid-proximal	67642	1642	0.089	0.060	-0.192	-0.049	0.008	-0.013	0.271
Midshaft	102208	1590	0.125	0.052	-0.214	-0.014	0.002	-0.046	0.311
Mid-distal	93774	1767	0.109	0.058	-0.203	-0.053	-0.021	-0.087	0.303
Distal	2276	1741	0.047	0.018	-0.079	-0.002	0.000	-0.001	0.115
Posterior									
Proximal	123907	1191	-0.025	-0.018	0.051	0.010	0.003	0.017	0.075
Mid-proximal	238875	1655	-0.067	-0.047	0.150	0.012	-0.026	0.052	0.214
Midshaft	100989	1635	-0.100	-0.067	0.196	0.025	-0.033	0.027	0.285
Mid-distal	94452	1623	-0.070	-0.057	0.156	0.004	0.028	0.087	0.233
Distal	86165	1725	0.009	-0.011	-0.002	-0.004	0.038	-0.014	0.039
Medial									
Proximal	66935	1448	0.055	0.041	-0.111	-0.025	0.146	-0.062	0.211
Mid-proximal	112946	1830	0.076	0.049	-0.159	-0.019	0.061	-0.009	0.229
Midshaft	22786	1892	0.068	0.047	-0.141	-0.020	0.032	0.015	0.202
Mid-distal	93732	1848	0.075	0.051	-0.148	0.001	0.012	0.000	0.212
Distal	11522	1822	0.047	0.025	-0.098	-0.011	-0.054	0.030	0.147
Lateral									
Proximal	123797	1256	0.014	0.015	-0.036	-0.009	-0.002	-0.049	0.067
Mid-proximal	6052	1721	-0.023	-0.007	0.039	0.004	-0.015	-0.028	0.062
Midshaft	101465	1792	-0.059	-0.026	0.099	0.004	-0.008	0.030	0.147
Mid-distal	93738	1856	-0.024	-0.023	0.082	0.021	0.015	0.076	0.126
Distal	86766	1830	-0.012	-0.009	0.025	-0.003	-0.010	0.009	0.037

### Juvenile tarsometatarsus: morphological comparison and bone densities

**Fig 8:**
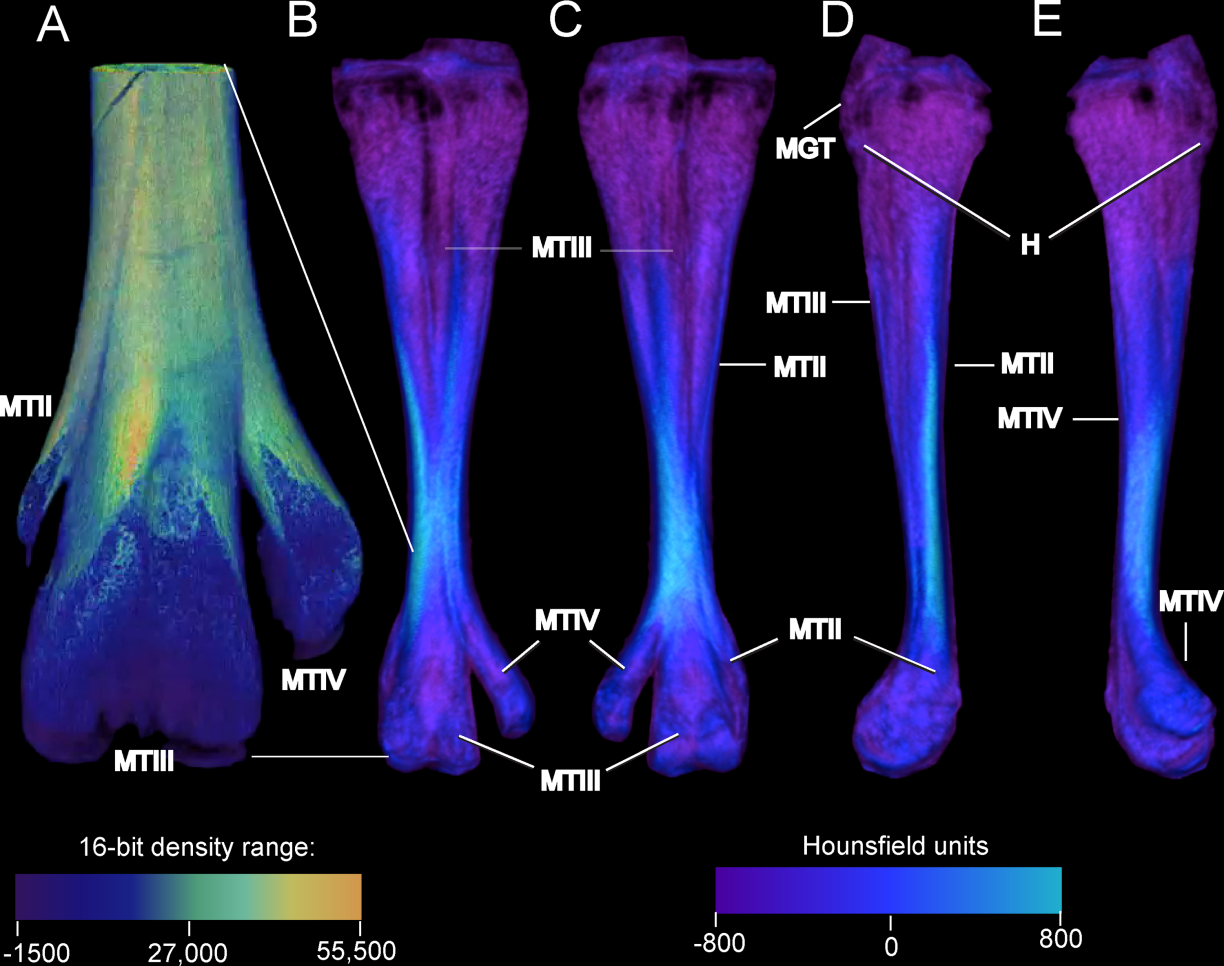
Densities on the external surface of a juvenile ostrich left tarsometatarsus reconstructed to show detailed anterior morphology (A), and anterior (B), posterior (C), medial (D), and lateral (E) complete views. Note the underdeveloped hypotarsus and undefined intercodylar fossa, and that MT III is discrete and visible anteriorly (A) for half the length of the tarsometatarsus. Densities are uniformly low (B-E), particularly at the with cancellous bone and calcified cartilage at the proximal and distal ends. Abbreviations: H = hypotarsus, MGT = M. gastrocnemius tendon. MT II = metatarsal II, MT III = metatarsal III, MT IV = metatarsal IV. The density color scales show (A) the midpoint and depicted range of the 16-bit total range micro-CT scanner, and (B-E) of Hounsfield units (HU) in a medical-grade scanner. The micro-CT scanner resolves finer gradations of density, and output of the lower-resolution medical scanner requires a restricted color scale to show the depicted morphology.

**Fig 9:**
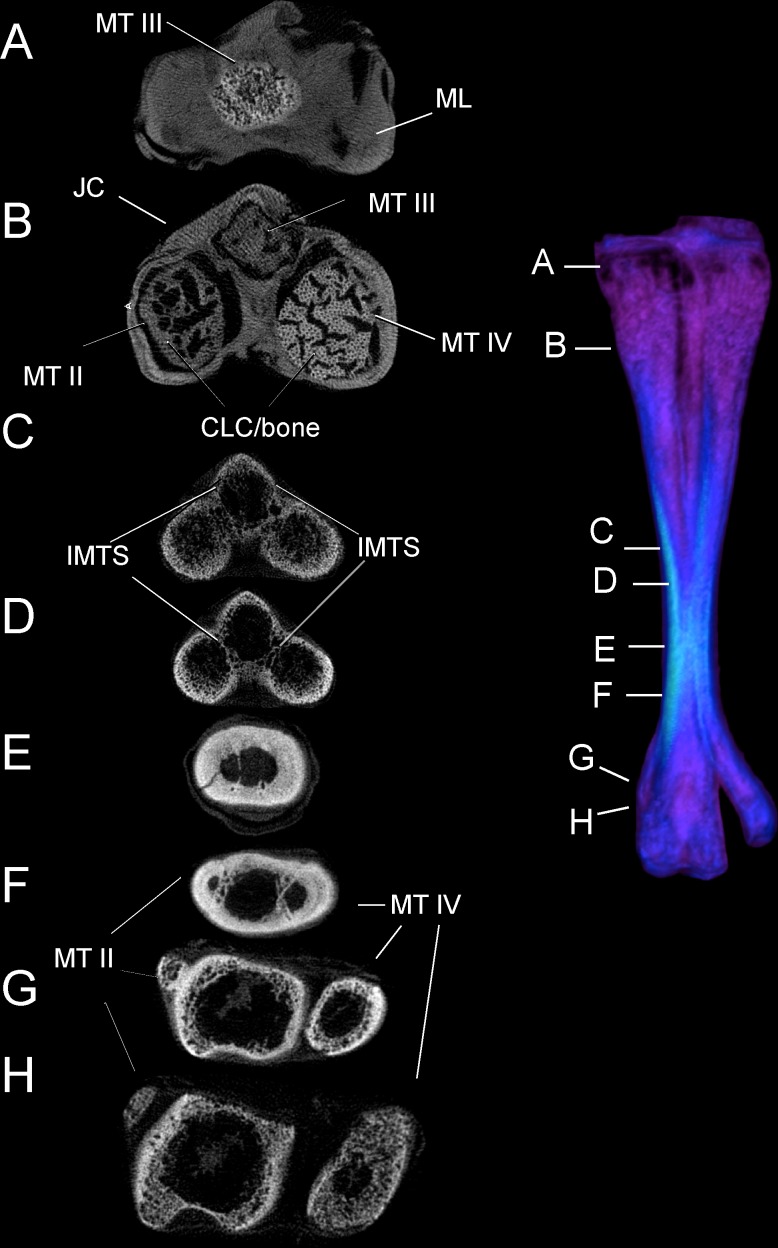
Cross-sections through a juvenile tarsometatarsus, from proximal (A) to distal (H). Most abbreviations are as in Fig 6; CLC-bone indicates a region where calcified cartilage is being replaced by bone, with many blood vessels perpendicular to the section. Only a small region of the tarsometatarsus has extensive fusion and compact bone (E and F). Note that the second metatarsal (MT II) is still prominent at this age, evident in F, G, and H.

In contrast with an adult, the juvenile ostrich tarsometatarsus (Fig 8) is relatively more flared and broad at the proximal end. The juvenile exhibits an underdeveloped hypotarsus, and the intercondylar fossa is virtually absent. The juvenile TMT’s diaphysis is significantly shorter relative to the element’s width, and ankylosis of MT II, III, and IV is more clearly observed on the external surface in the juvenile. CT imaging reveals internal tripartite division of metatarsals II, III, and IV throughout the entire length of the element in the juvenile (Fig 9). This is not seen in the adult, though present proximally, this division is lost before the midpoint of the shaft. In most adults, both internal and external evidence of MT II is lost after the midpoint of the shaft. A small spur of MT II is present in some adult specimens. This contrasts with the juvenile, as MT II splits from the main shaft and terminates as a vestigial but distinct element at the point where MT III and IV begin to flare into the articular condyles. Extensive cartilage is present in the juvenile tarsometarsus in both proximal and distal articular ends. Compared to the adult, the juvenile tarsometatarsus is considerably less dense, with extensive cartilage, less densely ossified compact bone, and relatively more cancellous bone (Figs 7 and 9).

### Finite element results

#### Proximal region

**Fig 10:**
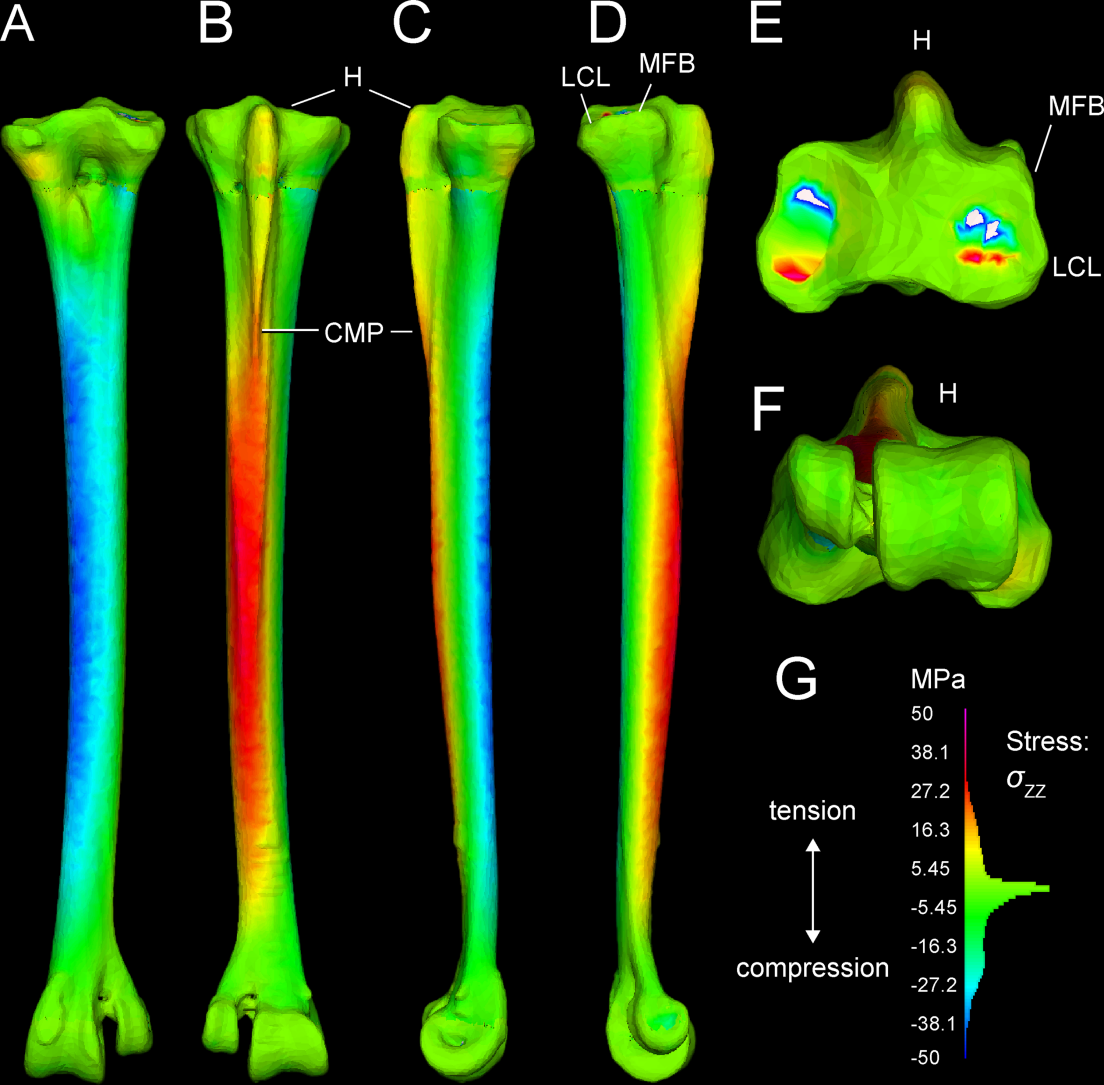
*σ*_*ZZ*_ stresses in the ostrich tarsometatarsus reflect bending along its proximodistal axis (Z-axis of the user-specified coordinate system). The scale (lower right) is a histogram of the proportion of elements experiencing different magnitudes of stress, divided into 100 levels between +/-50 MPa. Note that most elements have low *σ*_*ZZ*_ magnitudes, close to 0 MPa. A (anterior), B (posterior), C (medial), and D (lateral) views reveal anteromedial compression and posterolateral tension. E and F illustrate artifactually high stresses proximally, and realistically low stresses distally at the ground reaction force. G. Hot (red-violet) colors indicate tension, and cool (blue) colors indicate tension. Note moderate, distally diminishing tensile stresses on the hypotarsus from extensor forces (B, C, D), and low tensile stresses at attachment of M. fibularis brevis (MFB) and Ligamentum collaterale laterale (LCL).

**Fig 11:**
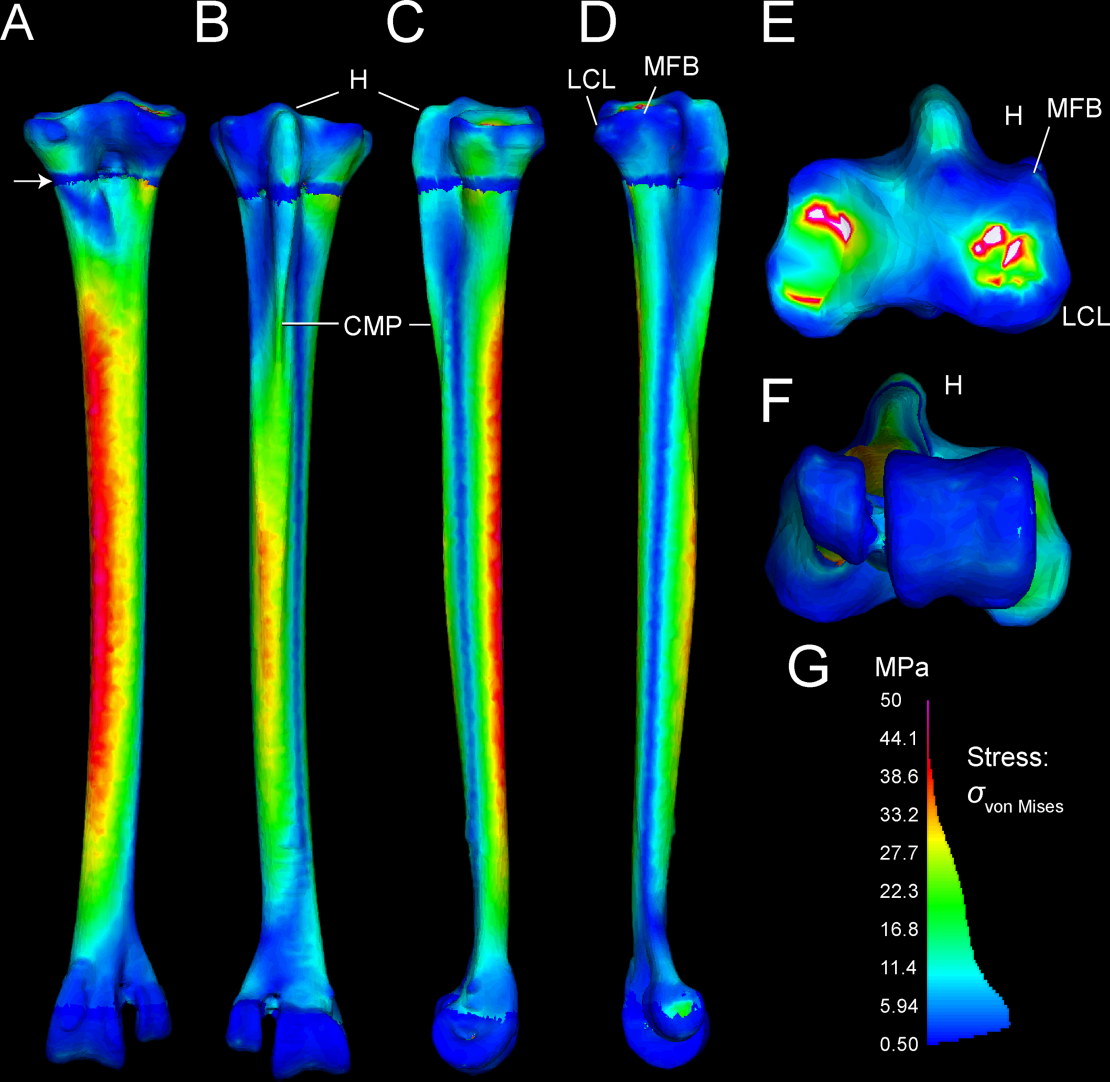
*σ*_von Mises_ stresses in the ostrich tarsometatarsus represents the maximum strain energy distorting a given element, and indicates proximity to breaking. A (anterior), B (posterior), C (medial), and D (lateral) views reveal the highest stresses in the shaft concentrated anteromedially. A white arrow (A) indicates the proximal transition between cancellous and compact material properties. Muscle induced stresses are evident on the hypotarsus (small H), continuing onto the Crista plantares medialis (CPM); however, these are low compared to stress in the main tarsometatarsus shaft. E. and F. show clipped stresses (white as with clipped highlights in photography; greater than the maximum in the color scale) at the proximal constraints, and low stresses distally. G. The *σ*_von Mises_ color scale from 0–50 MPa runs from low (blue) to high (red and pink). As in Fig 10, the scale also depicts a histogram of the proportion of elements experiencing different stress magnitudes. The majority of elements have low *σ*_von Mises_ magnitudes between 0 and 20 MPa.

**Fig 12:**
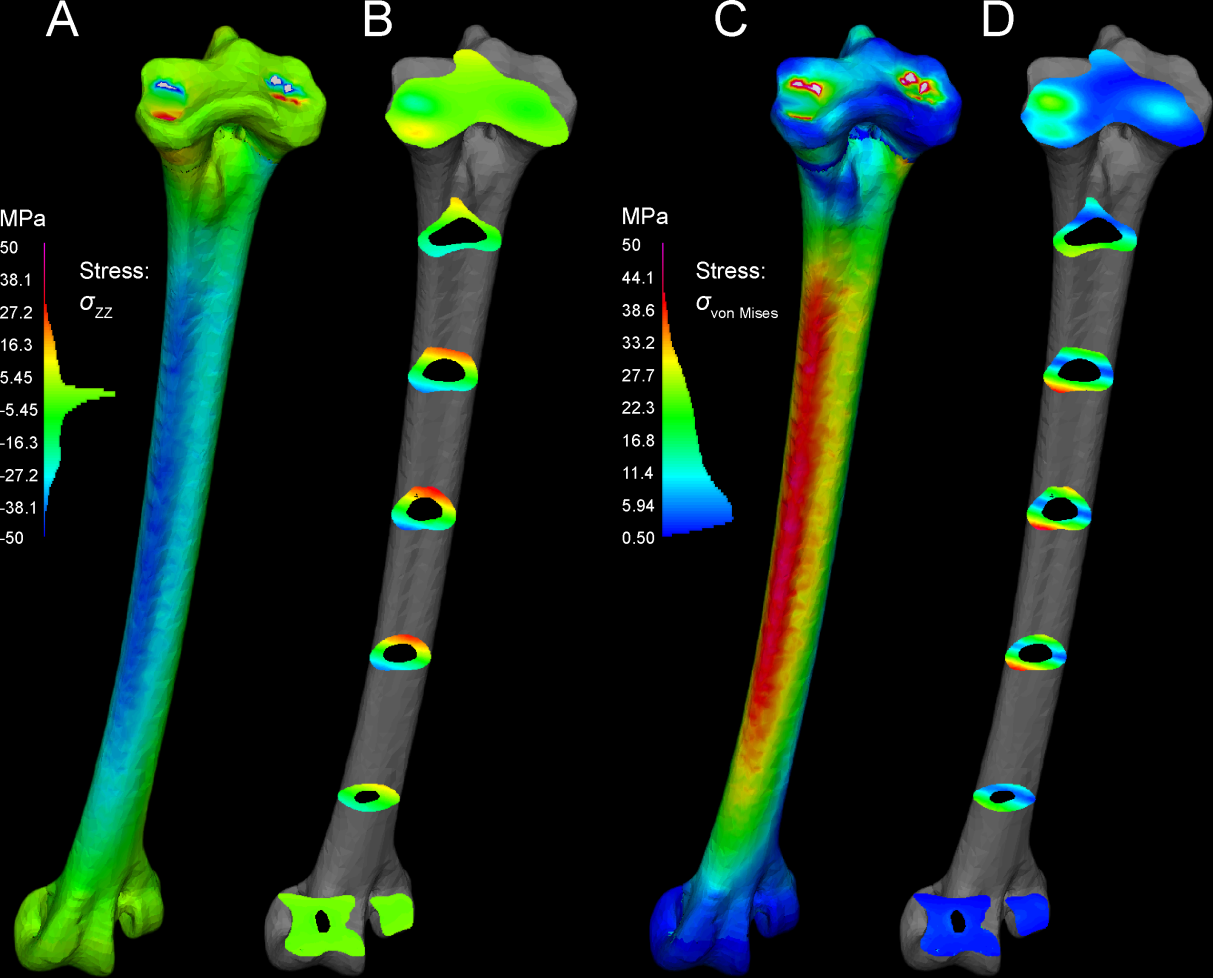
Cross-sections reveal internal stresses in the tarsometatarsus. Stress color scales and histograms are the same as in Figs 8 and 9. A. An oblique view of tarsometatarsus shows external *σ*_*ZZ*_ stresses, B. Cross-sections indicate regions of low *σ*_*ZZ*_ stress (green), tension (“hot” colors) and compression (“cool” colors). C. Another oblique view depicts external von Mises stresses. D. Cross-sections show distribution of low (blue), intermediate (green), and high (yellow and red) *σ*_von Mises_. Note that in this scale, with maximum absolute values of 50 MPa, stresses are clipped out at the constraints on the mesotarsal joint, appearing white because their values are higher than the highest stresses in the color scale. The restricted scale allows us to better visualize stress differences, with greatly contrasting colors for moderately different stresses. The highest stresses are medial (D), and are compressive (B).

Tables 3 and 4 present stresses and strains from forces simulated as experienced during a fast run, at discrete anatomical features (Figs 1 and 2) and at locations along the tarsometatarsus shaft (Fig 6). Fig 10 differentiates compression and tension stresses primarily along the long axis of the metatarsus (*σ*_*zz*_), and Figs 11 and 12 depict von Mises stresses. Proximal values are near constraints, and must be interpreted cautiously. Artificial peaks of von Mises stress *σ*_*vm*_ and strain *ε*_*vm*_, up to 291 MPa and 5.91%, respectively, where cancellous and compact bone come together in our model, which we consider to be an artifact of assigning material properties. Stress is moderate otherwise, even at the proximal constraints against long-axis displacement (Figs 10–12). Stresses diminish rapidly away from the constraints; for example, a node at the Cotylus medialis experiences *σ*_*vm*_ of 5.03 MPa and *ε*_*vm*_ of 1.01%. Close proximity to constrained nodes gives fairly high stress and strain at proximal entheses. Von Mises strains at the attachments of M. gastrocnemius to the hypotarsus (*ε*_*vm*_ = 2.05%) and of M. fibularis brevis (*ε*_*vm*_ = 1.85%) approach maximum safe values for cancellous bone (1.65–2.11%; Keaveny [28] reports these and lower values). However, stress and strain become much lower deep to the surfaces of these attachment sites (Fig 12), and tendon-to-bone gradations within muscle attachment enable greater strains than bone can withstand alone. *σ*_*vm*_ stresses on the hypotarsus, with a strong pull by M. gastrocnemius, are in the range of 10 MPa.

#### Main tarsometatarsus shaft

On the tarsometatarsus shaft distal to “mid-proximal” (Fig 6), most compressive stress occurs anteromedially (Figs 10 and 12A and 12B: negative *σ*_*ZZ*_, cool colors), and tensile stresses posterolaterally Figs 11 and 12A and 12B: positive *σ*_*ZZ*_, hot colors) under the current loading regime. In the anteriomedial region, *σ*_*vm*_ and *ε*_*vm*_ are slightly higher than they are posterolaterally (16–34 MPa and 0.1–0.31%, versus 3–31 MPa and 0.04–0.29%, respectively: Tables 1 and 2). By the *σ*_*vm*_ yield criterion, safety factors range from four to five in regions primarily under either tension or compression. For example, the posterolateral *σ*_*vm*_ maximum of 31 MPa is 27% of a conservatively low tensile yield stress of human compact bone, and the anteromedial *σ*_*vm*_ maximum of 34.1 MPa is 19% of the compressive yield stress.

#### Distal stresses and strains at *F*_*GRF*_ and phalangeal flexor and extensor application

Stress and strain are relatively low distally at the condyles of MT III and MT IV, where the ground force is applied. *σ*_*vm*_ ranges here from 0.76–2.34 MPa, and *ε*_*vm*_ from 0.15–0.47% (Tables 1 and 2: Trochlea metatarsi III lateral and medial: TM III lat. and med.; intermediate midpoint of Trochlea metatarsi IV: ITM IV). Safety factors are between two and ten for simulated values of *ε*_*vm*_ compared with yield values [28,29], and much higher compared with values at which cancellous bone becomes non-functional (*ε*_ult_* in Table 4; [28]).

#### Stresses and strains with variants of muscle attachments

**Fig 13:**
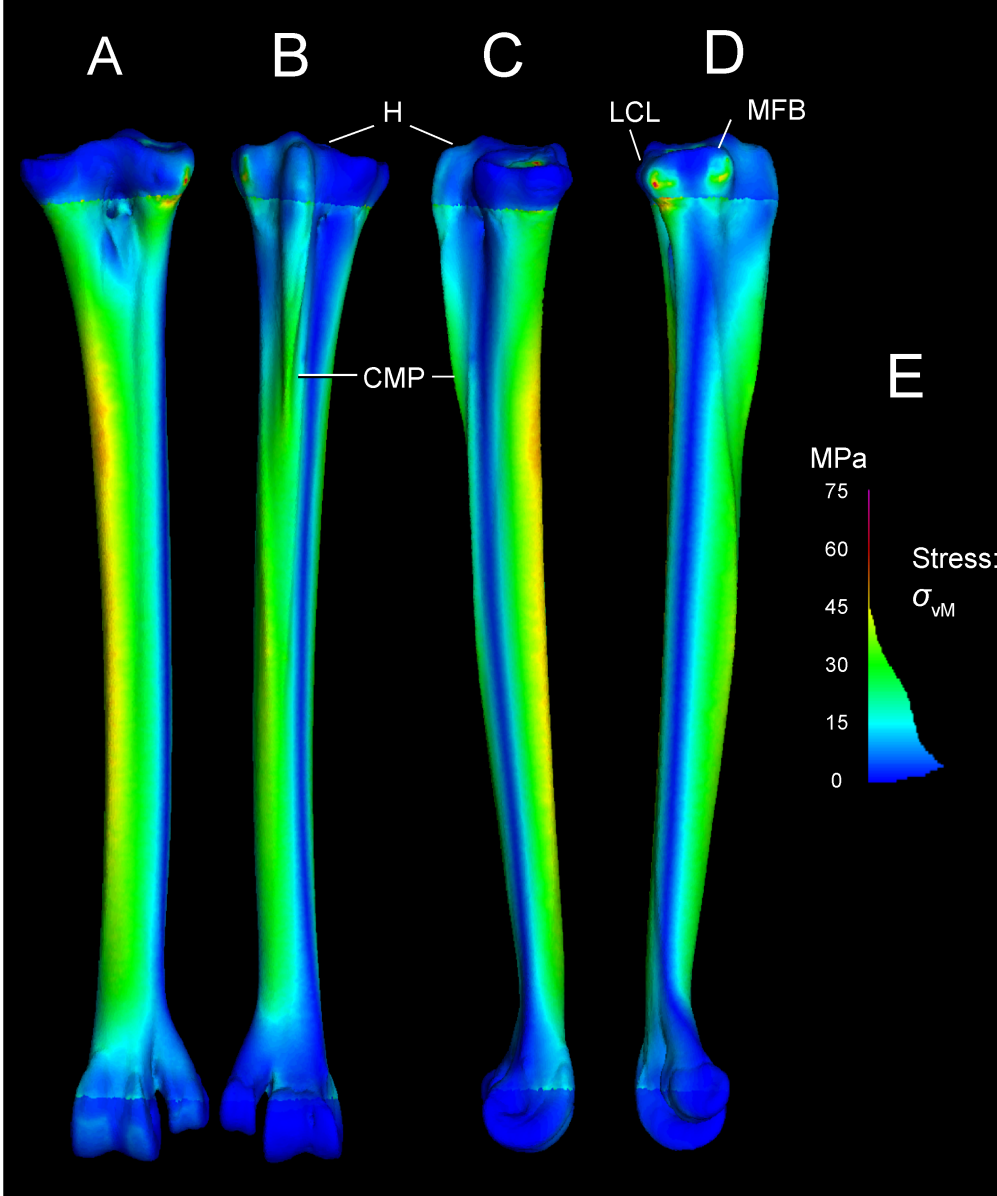
von Mises stresses in the *Struthio* tarsometatarsus with m. gastrocnemius restricted to the posterior insertions and excluded from the hypotarsus, and m. fibularis longus inserting alone on the hypotarsus. The Fig depicts A (anterior), B (posterior), C (medial), and D (lateral) views. Peak stresses at the constraints of 76 MPa (E for scale) are lower than if all forces necessary to counteract the *F*_*GRF*_ moment are applied to the hypotarsus.

Fig 13 depicts a loading scenario (very likely with most dissection descriptions of *Struthio*) with separate muscle attachments for ankle extension, through the M. gastrocnemius tendon only inserting posteriorly on the tarsometatarsus, and M. fibularis longus inserting alone on the hypotarsus. This loadcase distributes the force between the muscles, reducing the force at either attachment. This array of muscle insertions trivially decreases peak von Mises stresses on the TMT at the proximal constraints, although stresses in the distal part of the TMT shaft are similar to those of the main analysis.

**Fig 14:**
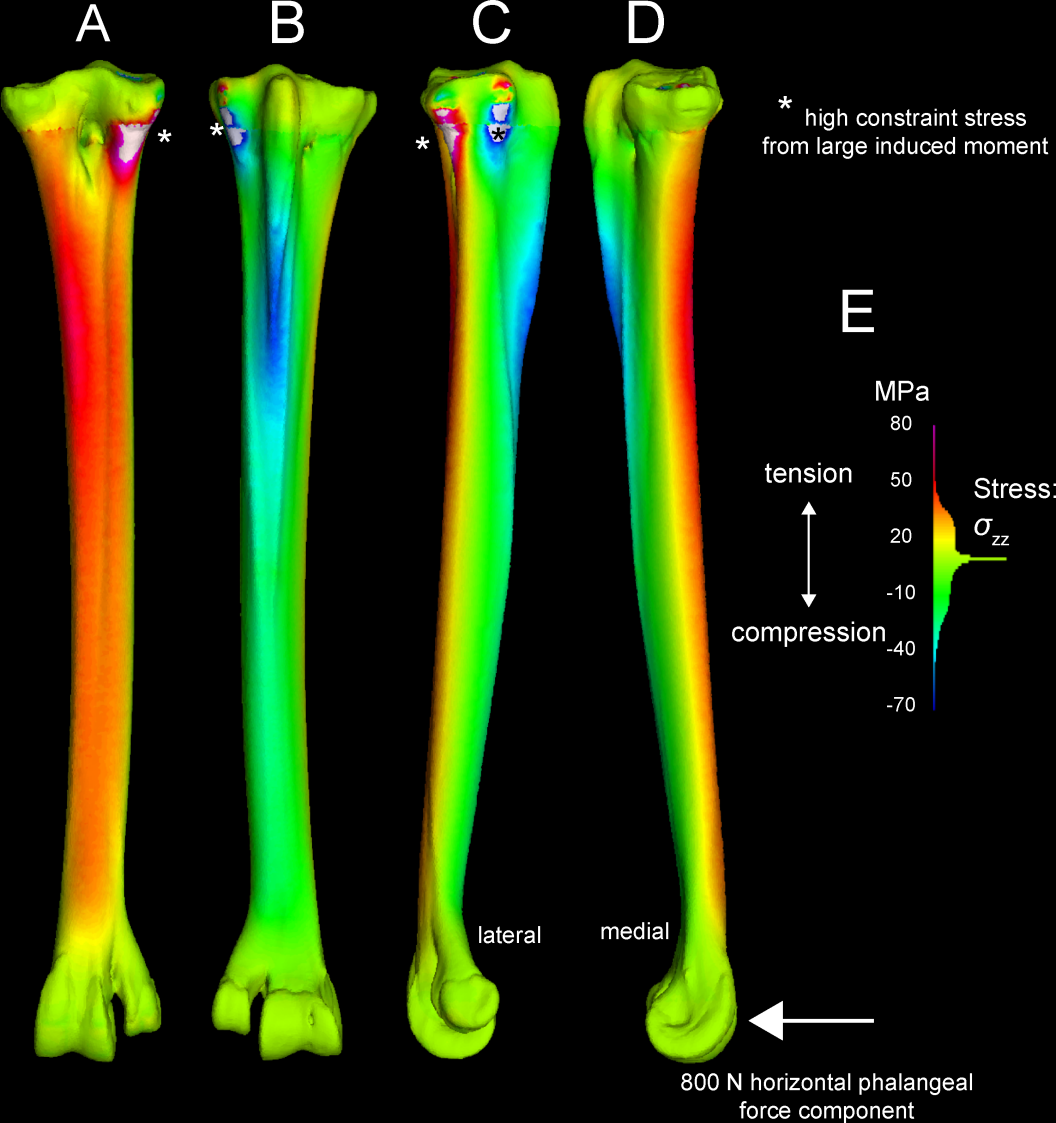
An exploratory large force of 800 N is applied posteriorly to the articular condyles of the *Struthio* tarsometatarsus, causing tension anteromedially and compression laterally. Especially high stress at the constraints (*) suggests too great a force magnitude, causing a very large moment about the constraints.

Another variant on muscle forces in the original analyses simulates a high posterior force on the TMT from the digital flexors pulling back on the phalanges. A high, 800 N force from the digital flexors increases tension anteriorly and compression posteriorly on the TMT (Fig 14), the opposite of the pattern with ankle extensors alone.

#### Convergence analysis

**Fig 15:**
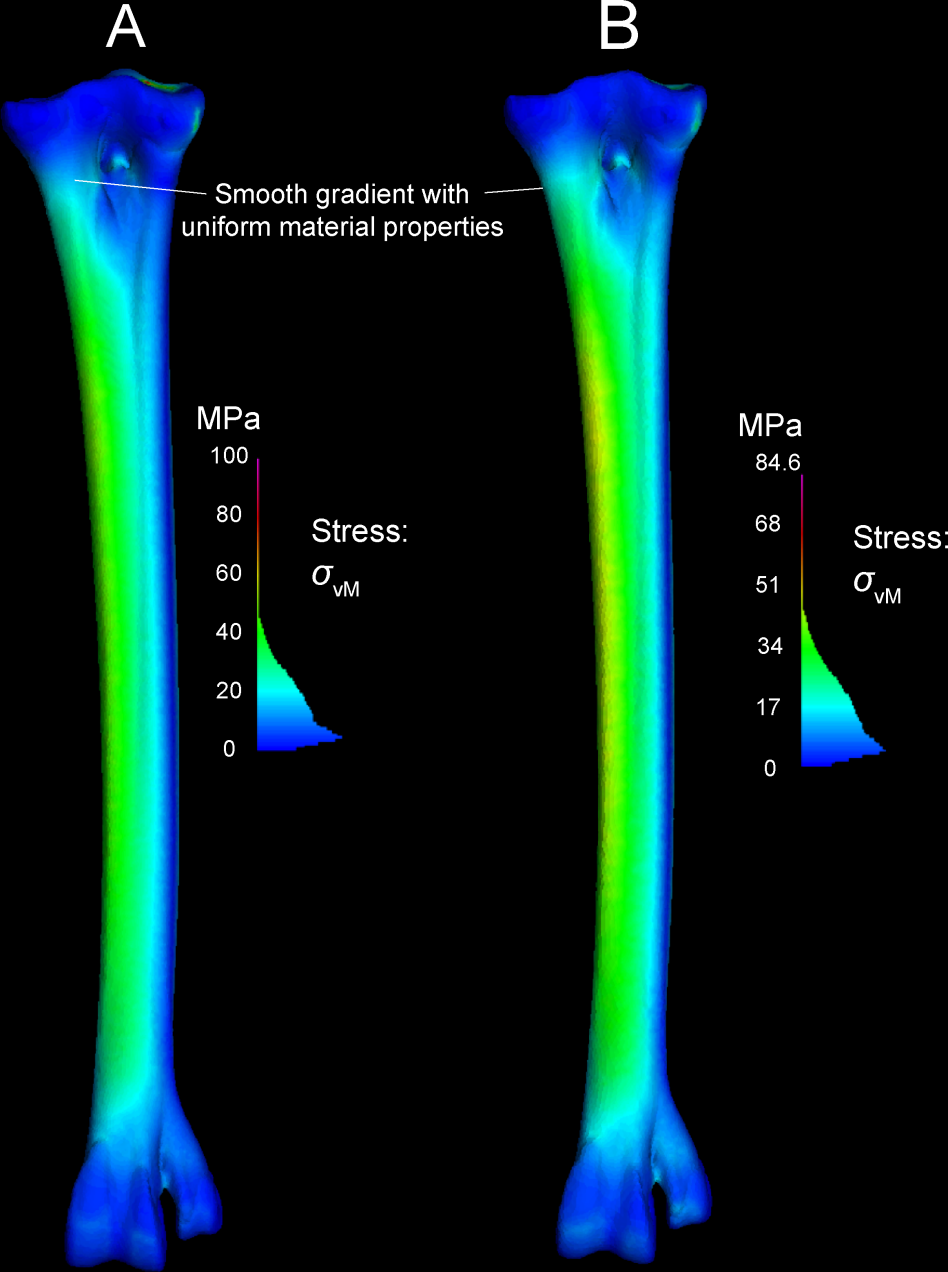
Convergence and material property sensitivity in highest (A) and lowest (B) resolution models. The lowest resolution model (77,722 nodes) has lower peak von Mises stresses (84.6 MPa) than the highest resolution model (180,029 nodes; 100 MPa). Peak stress occurs at the proximal constraints in both models. Stresses elsewhere in the models are similar. The lower peak value in the stress color histogram (B) makes anteriomedial stresses appear greater (yellower) than in the high resolution model, but sampled stresses are nearly identical throughout the TMT shaft. Both models have material properties of compact bone. Note the smoother transition from proximal to distal stresses than in models with both cancellous and compact bone properties in appropriate regions (Fig 11).

Away from the constraints, all models experienced peak von Mises stress of 50 MPa, plus or minus 2 MPa. The discrepancy of less than 5% between all models suggests that our initial model had sufficient resolution for further analyses. Fig 15 highlights the similarities, depicting the highest and lowest resolution models. There was no discernable pattern to the peak stresses at the constraints. As expected, the lowest resolution model (78,000 nodes; 385,000 tetrahedra) had the low von Mises stress at the constraints (84.6 MPa). However, the highest resolution model (180,000 nodes; 931,000 elements) had a lower constraint von Mises peak stress (100 MPa) than two intermediate-sized models. Because constraint stress and strain are artificially high, we do not attempt to interpret values at these positions (see above under “Interpreting finite element results” and the results below).

#### Effects of varying material properties on stress and strain

Fig 15 depicts models with compact bone properties applied to the entire element. Regardless of node and element numbers, these models had greater stress but lower strain proximally at the constraints, as expected from the much greater elastic modulus of compact bone compared to cancellous. However, the discrepancy was not as great distally at the force applications. The all-compact bone model also had much smoother results at the location where compact and cancellous material properties intersected in the multi-material model.

## Discussion

### FEA results are realistic distally, and will improve with automated material properties

Stress and strain magnitudes are unrealistic near constraints, and our model has an anomalous band of low stress (Figs 10–12) where compact and cancellous bone meets proximally (Fig 1; proximal to slice B in Fig 7). Saint-Venant’s principle [30,31] gives greater confidence in values farther from the proximal constraints, such as in the main shaft of the tarsometatarsus and distally near the application of reaction forces. Materially heterogeneous models potentially give more realistic results than homogeneous ones [31,32,33] (but also see [34]). One of our models has cancellous bone at the proximal and distal ends and compact bone elsewhere. Subsequent to our initial model construction, Eichenseer et al. [35] showed how a thin shell of plate elements with compact bone properties can be placed to surround the cancellous bone, which would be more realistic improvement to our model despite fairly low densities evident at these surfaces (Figs 1 and 2). A multi-material model with semi-automated assignment of elastic modulus [21,32,36] will likely improve on our results, especially with a smoother gradient between proximal cancellous and compact bone (Fig 11A).

### The initial mesh has adequate resolution to predict results of validation studies

Mesh characteristics also influence the likely accuracy of an FE model. Our convergence analyses revealed small differences in peak, non-constraint stresses (<5%) between our initial model and one with 1.6 times the number of nodes. Bright and Rayfield [32,37] investigate how well FEA approximates measured strains with varying mesh density, element size, and anatomical detail. The *Struthio* tarsometatarsus mesh consists of small (mostly ≤1 mm), four-node tetrahedra that Bright and Rayfield [37] report as good for approximating measured strain. In analyses of a pig cranium, Bright and Rayfield [32] discovered that FE results closely approach strain gauge results in flat regions of bone, and near force application. Conversely, results were less precise near finely contoured areas such as blood vessel grooves, and near constraints [32]. Based on these findings [32], we predict that the *Struthio* model will prove most consistent with strain gauge measurements along the tarsometatarsus shaft, and reasonably consistent with experimentally measured strains near an applied *F*_*GRF*_ [32].

### Tarsometatarsus densities and FEA: implications for ostrich locomotion

The adult *Struthio* tarsometatarsus has generally high density and stiffness of compact bone in the shaft of the tarsometatarsus, where it experiences the highest stresses. At a finer level, however, we found no specific correlations of density (as CT Hounsfield units) and stress or strain in the specimen’s compact bone. For example, regressing log10 HU against percentage *ε*_*vm*_ of sampled points in Table 2 yields an R^2^ of 0.07746. Assessing detailed, adaptive correlations between CT densities and stress will require a more complex material model, that indexes Hounsfield units of bone to Young’s modulus [18,19,20,21,36].

Even with only two material properties, *σ*_*vm*_ stresses indicate safety factors of 4–5 relative to yield stress, and 5–7 relative to ultimate stress (Table 3; [38]), in the tarsometatarsus shaft and distally at the tarsometatarso-phalangeal joints. This is consistent with ostriches running fast enough to exceed *F*_*GRF*_ of 2.5 times body weight. By the *σ*_*vm*_ stress yield criterion, bone strength and yield in the tarsometatarsus do not appear to limit speeds in adult ostriches (as with isometric muscle force: [39]). The greatest *ε*_*vm*_ strain values are closer to the *ε*_*yield*_ and *ε*_*ult*_ of compact bone (0.2–1%; [28,40]). Distal cancellous strains, however, have high safety factors relative to *ε*_*yield*_ or *ε*_*ult*_. Articular cartilage is highly viscoelastic, and excellent at resisting compression at high strain rates and protecting underlying bone. Cancellous bone in the distal condyles, deep to this cartilage, probably had greater safety factors than modeled here.

Our application of digital flexor forces, pulling the phalanges towards the tarsometatarsus, probably had too great a magnitude, inducing too great a moment on the TMT for realistic results (with a moment arm nearly as large as the entire TMT length). However, the reversal of the bending pattern (compare Figs 10 and 14) is enlightening. In life a lower force magnitude would reduce compression anteromedially and reduce tension (increasing compression) posterolaterally compared to the pattern evident in Fig 10. Because bone is stronger in compression than tension, a posterior component from digital flexors (and extensors) would increase safety factors in the ostrich TMT. When muscle firing patterns are unknown, exploring the effects of several hypothetical muscle force regimes can point the way to realistic picture of bone stress and strain in life.

### Biologically relevant refinements for future simulation

Anatomy, densities, and FEA results for the ostrich tarsometatarsus suggest specific improvements on our biomechanical modeling, beyond expansion of loading regimes [2,4,7,8,9,11] and material properties [21,36]. In particular, further consideration of force of the digital flexors will refine distally applied forces at the tarsometatarso-phalangeal joints. Studies of human and cow feet that incorporate soft tissues [41,42,43,44] will guide models that include cartilage, ligaments, tendons, and perhaps more informative constraints. Experimental validation, especially with fresh specimens and loadings at behaviorally realistic strain rates, will arbitrate the accuracy of the modeling and contribute inputs for refined analyses.

### Ontogenetic, selective, and evolutionary context of *Struthio* TMT functional morphology

Regardless of the n-th refinements possible with the FE model and its loadings, the descriptions of adult and juvenile tarsometatarsi in this paper are a stable and fundamental baseline for interpreting biomechanical results related to ostrich locomotion. CT-imaged internal structure and material densities are described here for the *Struthio* TMT for the first time, including fine density gradations in the juvenile specimen. Because ostriches grow so rapidly, these endpoints can anchor comparisons of ostrich anatomy and locomotion through their ontogeny, and investigations of selection and the ostrich TMT.

Bird species dependent on ground speed for locomotion rely on long legs and powerful muscles. Biometrics have been used successfully to explain ecological variability among species [45,46,47]. This study provides a novel means of studying form, function, and mechanics by combining FEA and CT data, useful for investigating the tarsometatarsus of *Struthio* or any other bird species.

The tarsometatarsus has high predictive power to infer behavior and ecological adaptation from morphology in birds [45,46]. Correlations of avian limb length to primary method of locomotion across broad groups have found that an elongated tarsometatarsus is not necessarily important in terrestrial environments [47]. However, an elongated tarsometatarsus does appear to be evolutionarily advantageous in *Struthio*. FEA revealed high safety factors, suggesting the tarsometatarsus has momentarily excessive construction [48] for ensuring sufficient foot strength when maneuvering to escape predators, delivering fatal kicks, or other less-usual but selectively critical demands.

Elongation of the tibiotarsus and tarsometatarsus compensates for limited femoral movement in birds, especially advantageous for fast running ground birds such as ratites. Ground reaction forces strongly load the femur in bending, which reduces the length of the element in cursorial birds [49]. This is reflected in ostrich morphology, as the femur is short and the tibiotarsus and tarsometatarsus are both elongated. This morphology results in a longer step length, hence increasing travel speed. An elongated tarsometatarsus may facilitate locomotion in their natural environment, as high speed would be ecologically advantageous when avoiding predators in a grasslands ecosystem.

Loss of the medial toe in *Struthio* is believed to reflect an evolutionary modification to reduce weight and increase speed. Our results of higher stresses on the anteromedial aspect of the distal tarsometatarsus may be a result of loss of the second digit.

CT data presented herein demonstrates that vestiges of an internal three-part division of the tarsometatarsus persists even in adults. Fusion and elongation of the TMT is supported by the fossil record in basal birds and closely related non-avian theropod dinosaurs (see Brusatte et al. [50] for a recent review). With both morphological and molecular analysis supporting a non-avian dinosaur origin for birds, modern cursorial birds offer a modern analogue to extinct theropod dinosaurs. Therefore, a detailed study of the tarsometatarsus in *Struthio* acts as a starting point for similar studies of extinct theropods.

## Materials and Methods

### CT scanning, imaging, and descriptive anatomy

The ostriches used in this study were salvaged from commercial breeders after dying from natural causes. They were donated to the University of Calgary Museum of Zoology in Calgary, Alberta and the Ohio University Vertebrate Collections in Athens, Ohio. No animals were harmed or sacrificed for the purpose of this research.

The tarsometatarsus, tibiotarsus, and femur of an adult ostrich (UCMZ) were CT scanned on a GE Lightspeed scanner (Canada Diagnostic Centres, Calgary, Alberta), at 140 kV and 175 mA, with 1.25 mm spacing and 1.25 mm overlap. A small juvenile ostrich (UCMZ) was scanned on a NewTom 3G orthodontic scanner (Aperio Services, Verona, Italy), at 110 kV and 6.19 mAs; slice thickness was 0.5 mm. Its tarsometatarsus was dissected, removed, and scanned on a SkyScan 1174 compact micro-CT, at 50 kV at a resolution of 20 μm. The juvenile specimen was placed in a carved depression in a block of low-density foam, which was then secured to the scanner’s rotating stage. Because the juvenile’s tarsometatarsus is taller than the SkyScan’s detector, it was scanned twice with respective proximal and distal ends placed in the foam.

To evaluate internal anatomy, CT scans were read as DICOM data into OsiriX, and evaluated as slice data and volumetric reconstruction. In the 3D reconstructions, the tarsometatarsus data was isolated from other elements with the scissor tool. We visualized densities with the NIH color palate, which facilitates density comparisons better than a grayscale spectrum. Internal structures, external osteology, and soft tissue attachments of the adult specimen were described with reference to the literature. The terminology concurs with the Nomina anatomica avium [51] with supplementary information provided by Gangl’s studies [6,52].

### Finite element geometry and meshing

CT data for the adult *Struthio* tarsometatarsus were exported as DICOM series into Avizo (VSG, Burlington MA, USA), and surface meshes constructed. The initial surface mesh consisted of 3 million triangles, many of which had high aspect ratios which introduce numerical errors and artificially high strains [52] into FE simulations. Ideally all triangles will be isometric, but aspect ratios of 10–1 or less are adequate for accurate results. Correcting all aberrant triangles in a 3-million triangle surface mesh is time- and computationally prohibitive (often crashing the program when it attempts to correct intersecting triangles). To streamline the process and ensure adequate resolution, surface meshes were reduced to 50,000 triangles without loss of anatomical detail, and triangle aspect ratios reduced to 10-to-1 or less. These high-quality triangles were then subdivided to produce many more, smaller elements across the surfaces (“Refine faces” in Avizo), which produces better-quality triangles than in the initial large mesh with equivalent triangle numbers. A few of the new, smaller triangles had high aspect ratios, which were again corrected to 10:1 or less. The small surface elements ensured that when solid meshed, the bones’ cortical walls and internal struts were modeled at least three elements deep, for accurate simulations of bending. The surface model was meshed in Strand7 (Strand7 Pty Ltd, Sydney, NSW, Australia) to produce an initial FE model of 112,630 nodes and 564,088 tetrahedral elements, with element sizes of approximately 0.8 mm internode distance. Although element numbers are typically reported for FE meshes, the number of nodes is a more realistic indicator of a model’s resolution and computational complexity. (High element numbers on their own are theoretically misleading; infinite elements can converge on a single node.)

The initial model has fewer elements than in many FE studies of vertebrate skulls. However, the tarsometatarsus is a geometrically simpler structure (closer in complexity to a single mandible), and our model has a comparable number of elements to an analyzed elephant femur ([33], although see [34]). Simpler structures require fewer nodes and elements; for example, simulation of a rectangular beam only three elements deep approximates bending stresses quite accurately.

To determine if our initial model had sufficient resolution to apply other loadcases for parameter-effect (sensitivity) analyses, we assessed convergence (how closely peak stress and strain results approximate those of a model with infinite resolution) by constructing a model with 443,248 nodes and 2,086,152 elements. We constructed five further models with 77,722 nodes (385,953 tetrahedra), 125,246 nodes (636,995 tetrahedra), 151,947 nodes (779,940 tetrahedra), and 180,029 nodes (931,000 tetrahedra). If any lower-resolution model’s stresses at sampled points were within 5% of the highest-resolution model, we were confident to proceed with further loading regimes with the lower-resolution model.

### Material properties

We applied material properties appropriate to their respective locations: compact bone that occurs in the main shaft of the tarsometatarsus, and cancellous bone that occurs proximally and distally. Assigned material properties (Table 1) are those of compact and “bulk” cancellous bone (not of individual trabeculae, which have a stiffness closer to that of compact bone: [53,54]). Appendicular compact bone is orthotropic, with the highest elastic modulus parallel to the long axis. To the long axis of the element, we applied the elastic modulus *E*_*zz*_ of emu (*Dromaius novaehollandiae*) femoral cortical bone under high strain rates [55] (15.86 GPa). Both emu and ostrich appendicular bone has *E*_*zz*_ of 13–14 GPa under lower strain rates [55]. Using the high-strain-rate *E*_*zz*_ value for emu is appropriate because bone stiffness is load-rate dependent, and loads from fast running would introduce high strain rates. Using an elastic modulus collected at high strain rates introduces some realism to a linear, quasi-static analysis, which necessarily simulates the load as steady-state. We assume that *E*_*zz*_ a running ostrich’s appendicular bones would be similar to that of emu, as they are at lower strain rates. Longitudinal modulus was multiplied by 0.57 to obtain both transverse moduli [39]. In the absence of data for ratites, assigned shear moduli were those of human compact bone as determined through ultrasound experiments [56]. Poisson’s ratio of the compact bone was set to 0.3 [57]. Cancellous bone was considered isotropic, with an elastic modulus of 0.64 GPa and Poisson’s ratio of 0.29 [58,59].

### Finite element simulations: boundary conditions

**Fig 16:**
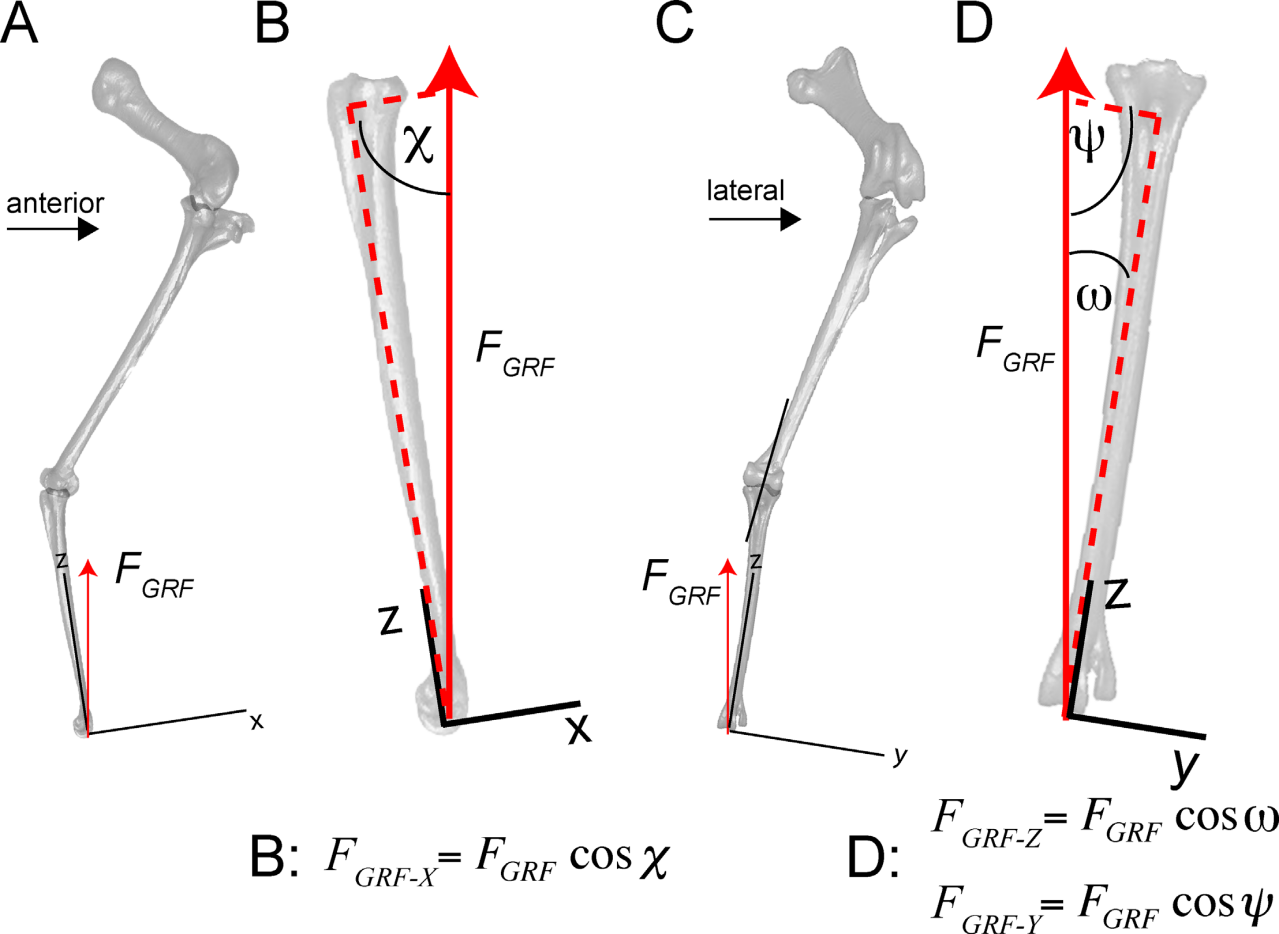
Limb posture, local coordinate system, forces, and components of the ground reaction force (*F*_*GRF*_) are incorporated into finite element (FE) simulations. *F*_*GRF*_ is applied to the distal end of the tarsometatarsus. A. Lateral view (left side, reversed) of ostrich femur, tibiotarsus+fibula, and tarsometatarsus (from top to bottom). B. Close-up of the tarsometatarsus, depicting resultant *F*_*GRF*_ and angle χ (81.327°) for computing its x-axis component *F*_*x*_ (left equation). C. Anterior view of ostrich leg. D. Close-up of the tarsometatarsus in anterior view, showing the resultant *F*_*GRF*_ and angles ψ (82.102°) and ω (5.006°) used for calculating y and z components, respectively (center and right equations). In these views the bones appear slightly shorter than their true lengths, because they are angled from the vertical.

**Fig 17:**
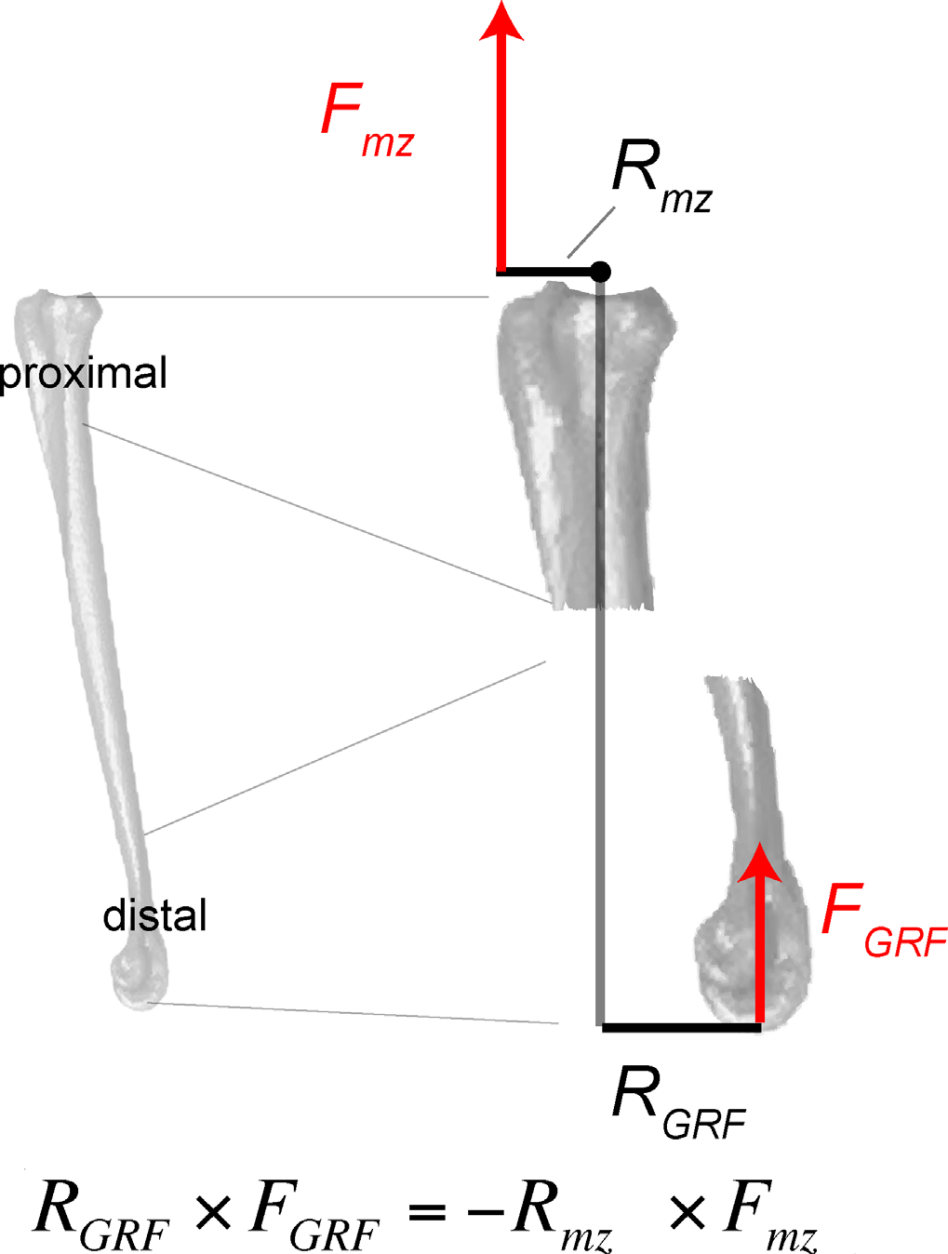
Quantities necessary for determining the extensor force, *F*_*m*_ of M. gastrocnemius, include forces *F*, moment arms *R*, and angles (Fig 16) relative to the tarsometatarsus coordinate axes. Moments about the center of rotation (proximal dot) must equal 0. The ground reaction moment *R*_*GRF*_ x *F*_*GRF*_ is therefore equal and opposite to a balancing extensor moment *R*_*mz*_ x *F*_*mz*_. When the vertical muscle force *F*_*mz*_ is calculated, the resultant *F*_*m*_ and its x and y components can be determined by the law of cosines [50] (Figs 15 and 16; Table 5).

**Fig 18:**
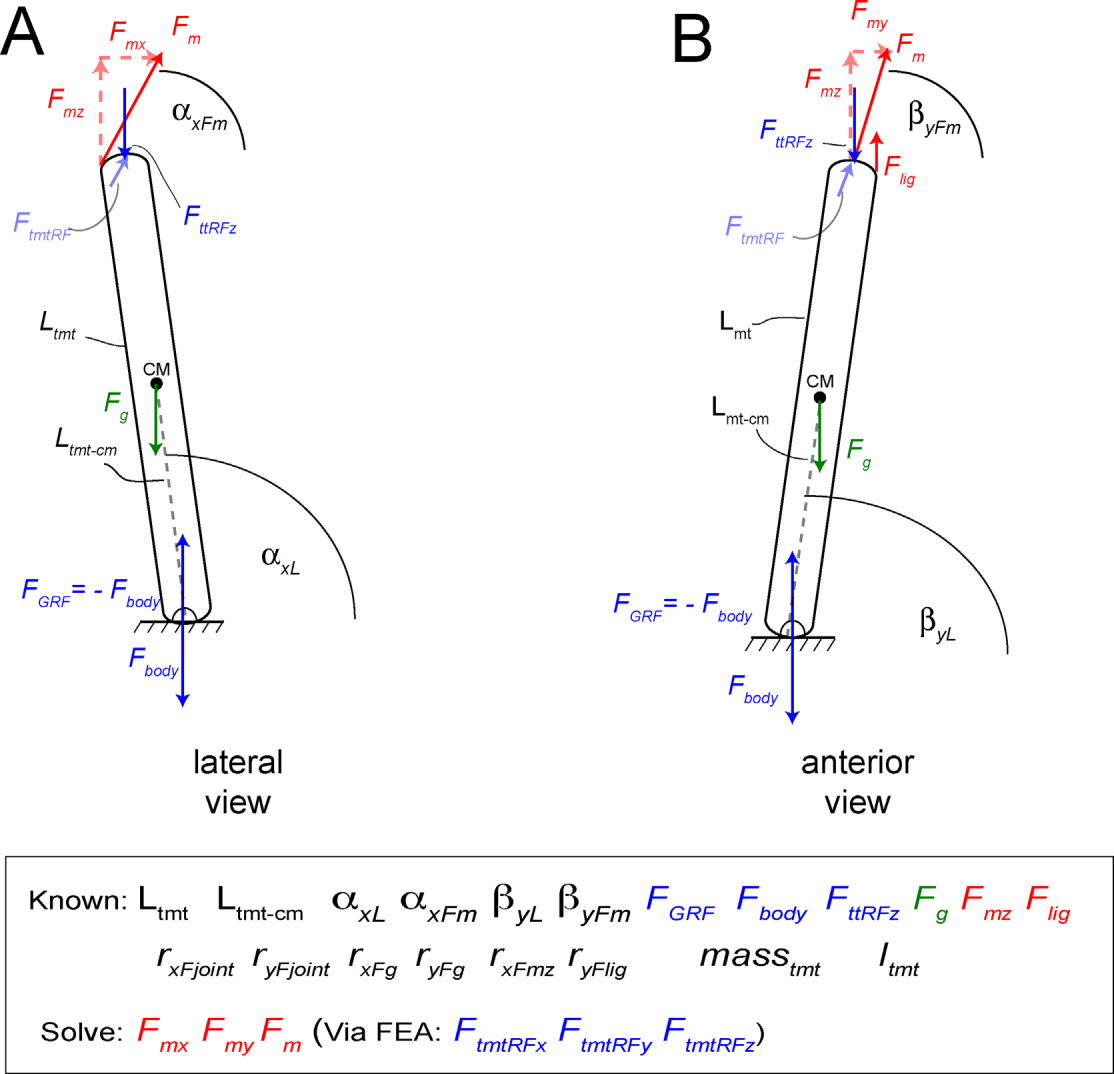
Problem statement diagrams of the tarsometatarsus in lateral (A) and anterior (B) views, for determining component and resultant values of extensor force by M. gastrocnemius (*F*_*m*_). Angles are defined with other variables in Table 4, and listed in Table 5. Components of *F*_*m*_ in the global and anatomical coordinate systems are listed in Table 6. FEA can determine many quantities listed as “Known”, including *F*_*lig*_ at the ligamentous constraints, and tibiotarus and tarsometatarsus joint reaction forces *F*_*ttRF*_ and *F*_*tmtRF*_ at the constrained proximal surface of the metatarsus. Moment arms r will vary with angles through the motion, producing moments when coupled with their forces; the FE simulations incorporate these quantities at one position. Gravitational force *F*_*g*_ and its moments on the tarsometatarsus cm (incorporating mass_tmt_ and mass moment of inertia *I*_*tmt*_) contribute trivially, compared to effects of muscular, ground, joint forces.

Constraints are necessary in FEA to prevent rigid body motion and allow deformation of a structure. However, fully fixing all nodes against motion in x, y, and z is unrealistic, and finite element simulations can give artificially high stress and strain results at constrained nodes. We assumed that the tibiotarsus would constrain the tarsometatarsus from moving proximally (Z-axis of the universal coordinate system; Fig 16A and 16C; Fig 17; Fig 18), and that the lateral colateral ligament and M. fibularis brevis constrained it from slipping laterally. Varying the size of the constraint area resulted in trivial variation in stress and strain results.

**Table 5:** Problem statement variables used for determining ankle extensor muscle forces, in Figs 14–16 and Table 6.

Known:	
*Ltmt*	tarsometatarsus length
*Ltmt-cm*	length from the point of rotation to the tarsometatarsus center of mass CM
*αxL1*	angle between the ground and the tarsometatarsus in lateral view, global reference frame
*αxFm*	angle between the ground and the resultant muscle force in lateral view
*βyL1*	angle between the ground and tarsometatarsus in anterior view
*βyFm*	angle between the ground and resultant muscle force in anterior view
*FGRF*	ground reaction force
*Fbody*	body force on the ground
*FttRFz*	reaction force of the tibiotarsus on the tarsometatarsus in the z-direction
*Fg*	Force of gravity on the tarsometatarsus center of mass
*Fmz*	z-component of the muscle force.
*Flig*	force from (FEA constraint) of ligament preventing sideways collapse of tmt
*rxFjoint*	moment arm of FttRFz in lateral view
*ryFjoint*	moment arm of FttRFz in anterior view
*rxFg*	moment arm of gravity on the CM in lateral view
*ryFg*	moment arm of gravity on the CM in anterior view
*rxFmz*	moment arm of Fmz. ryFlig = moment arm of Flig
*Mass*	mass of the tarsometatarsus
*Itmt*	Mass moment of inertia about the distal point of rotation
Solve for:	
*Fmx*	x-component of the muscle force
*Fmy*	y-component of the muscle force
*Fm*	resultant muscle force
*FtmtRFx*	x component of the tarsometatarsus reaction force upon the tibiotarsus
*FtmtRFy*	x component of the tarsometatarsus reaction force upon the tibiotarsus
*FtmtRFz*	x component of the tarsometatarsus reaction force upon the tibiotarsus

**Table 6:** Quantities and results for calculating tension *F*_*m*_ in the M. fibularis longus and M. gastrocnemius, by directional cosines [50] for components in the ground reference frame/coordinate system (ground c.s.), and rotation matrices [51] in the metatarsus’s reference frame/coordinate system in Strand7 (Strand7 MT c.s.). Angles are depicted in Fig 15, and defined in Table 5.

Angles in ground c.s.	*α*_*xFm*_	90—*α*_*xFm*_	*β*_*yFm*_	
(deg)	61.392	28.608	73.124	
Rotations about:				
y-axis:				
*α*_xFmxL1_—*α*_*xFm*_ (deg)	9.43			
Rotation matrix:	0.9864865	0	0.1638425	
	0	1	0	
	-0.1638425	0	0.9864865	
x-axis:				
*β*_*yL1*_—*β*_*yFm*_ (deg)	-5.92			
Rotation matrix:	1	0	0	
	0	0.9946669	-0.103139	
	0	0.1031398	0.9946669	
*F*_*m*_ (N)	z	x	y	Resultant
Ground c.s.	4201.655	2473.824	968.141	4971.017
Strand7 MT c.s.	3815.958	3141.492	529.623	4971.017

### Finite element simulations: forces

#### Ground reaction force *F*_*GRF*_ and phalangeal joint reaction forces

Simulations neglected inertial forces. Simulations were run under the assumption of linear behavior, in which stress and strain scale linearly with force magnitude, strains are small, and deflections do not change the stiffness of the structure. Under these assumptions, deflections, stress, and strain for any magnitude of load can be calculated by multiplying results of an initial simulation (even with unit forces) by the ratio of the desired load magnitude to the initially simulated force. We applied a baseline magnitude as the ground reaction force (*F*_*GRF*_) with a maximum magnitude of 2.5 times body weight, equivalent to a fast run. A regression of shank length to mass [60] yielded 100 kg for the specimen, and a maximum *F*_*GRF*_ of 2495 N.

Note that “*F*_*GRF*_” in this case is really the force of the proximal phalanges on the distal condyles of the tarsometatarsus. This requires several simplifying assumptions, clarifications about their effects, and two loadcases that account for forces across the tarsometarsus-phalangeal joints. Applying both of these loadcases (numbers 4 and 5 below) shows how unknown force transmission from the phalanges to the tarsometatarsus would affect stress in the TMT.

1. The proximal phalages support the tarsometatarsus vertically, so that their reaction forces on the tarsometatarsus are in line with the vertical *F*_*GRF*_ direction.
2. The true ground reaction force, from the subdigital pads through the body’s center of mass, requires contraction of the digital flexors to counteract extension of the phalanges.
3. In one set of analyses, forces from the digital flexors are simulated as contributing to the vertical reaction force on the TMT, and exert negligible non-vertical force on the TMT condyles. These forces would be incorporated into the 2495 N value for the reaction force, and not further calculated for the analysis.
4. An another set of analyses, we applied a posterior component to the phalangeal reaction force to simulate what would happen when digital flexors pulled the proximal phalanges towards the joint, in addition to their vertical contact. The forces are transmitted across the joint the anterior surfaces of these condyles.

For the analyses described in 4) above, an 800 N posterior load was applied, distributed proportional to the maximum isometric forces exerted by the flexors of digits 3 and 4 [10], seen in Table 7. These were 29% to digit 4 (231 N), and 71% to digit 3 (569 N). The 800 N is an arbitrary value, but useful to examine the sensitivity of results to such forces.

**Table 7:** Summary of applied forces for all finite element analyses, including components for the ground reaction force *F*_*GRF*_ and at muscle attachments.

Ground reaction force			Fx	Fy	Fz
			-343	376	2485
Functional group/muscle					
Ankle extension	*F*max	Proportion Fmax	Fx	Fy	Fz
*M*. *gastrocnemius-hypotarsus insertion*					
M. fibularis longus+M. gastroc.	4971	1	3141.5	529.6	3816
M. gastrocnemius (post. insertion)					4971
*Separate insertions*	*F*max [10]	Proportion Fmax	Fx	Fy	Fz
M. fibularis longus	1570	0.28	889	150	1079
M. gastrocnemius pars lateralis	1269				
M. gastrocnemius pars intermedia	552				
M. gastrocnemius pars medialis	2160				
M. gastrocnemius total		0.72			3579
Digit flexion (800 N applied)			Fx	Fy	Fz
Digit 3		0.71		-569.1	
Digit 4		0.29		-230.9	

**Fig 19:**
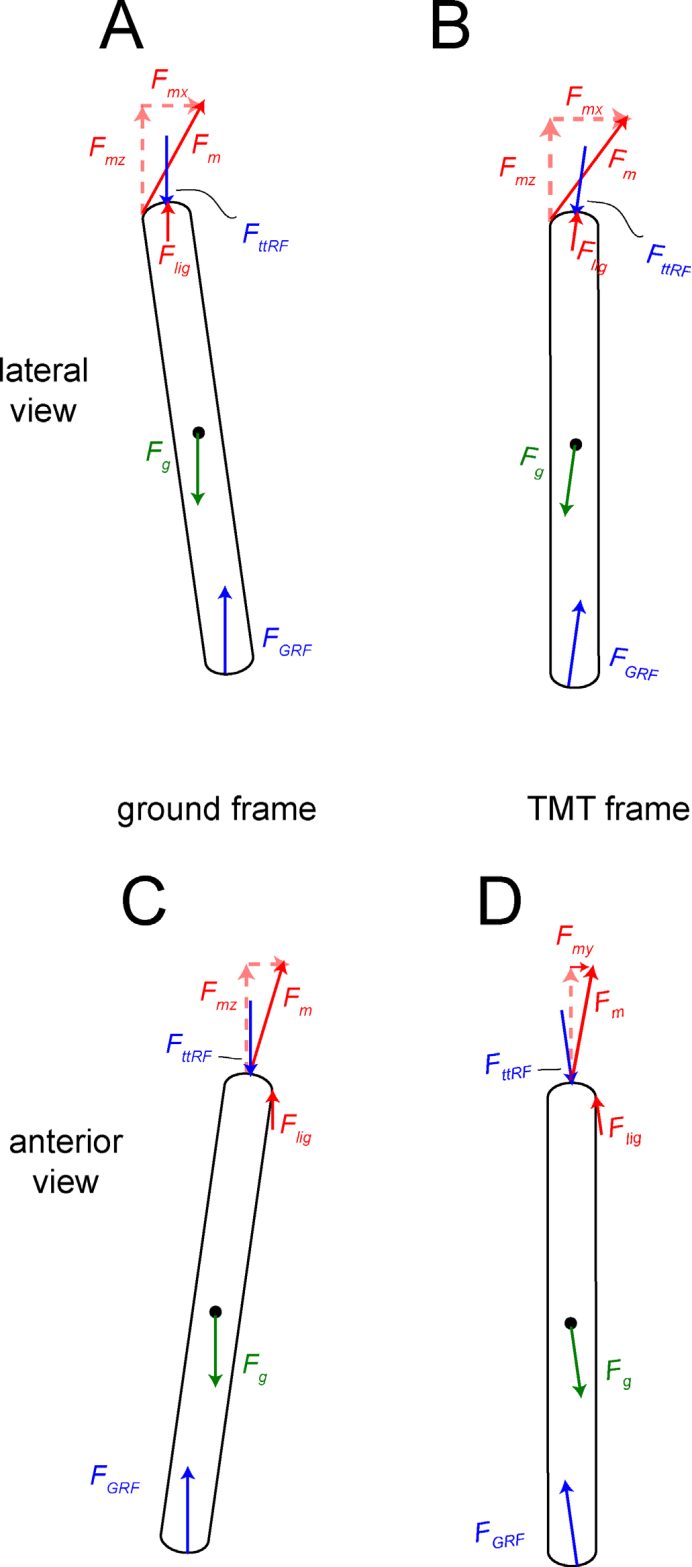
Free body diagrams for forces and their components acting upon the tarsometatarsus, including muscle and reaction forces as listed in Fig 15. A and B are for a lateral view, and C and D in anterior view. A and C are in the global reference frame and coordinate system of the ground (Fig 15). B and D are in the anatomical reference frame and coordinate system of the tarsometatarsus (TMT). Table 5 includes rotation angles and matrices for forces in this frame. Variable definitions are in Table 4.

Estimating force components was more complex than the magnitude of the ground reaction force. *F*_*GRF*_ at the midpoint of the step cycle in a straight run [11] is at its greatest magnitude and simplest direction (vertical), neglecting friction. We therefore graphically derived joint angles from kinematic data of Jindrich et al. [11], at a point in stance phase when the ground force is vertical. The local coordinate system for the tarsometatarsus was set with the x-y plane at the distal end of MT III, and z along the long axis. In this pose, the tarsometatarsus was inclined at 82.1° to the horizontal in the sagittal plane (lateral view), and 81.3° in the transverse plane (anterior view; Fig 19). The z-axis is at an angle of 5° from the vertical. The resultant GRF and ankle forces were therefore vertical in the universal coordinate system, but angled relative to the z axis of the local system. Their components in the universal x (anteroposterior), y (mediolateral), and z (proximodistal) directions were calculated with the law of cosines (Fig 10; [60]), and applied to the distal or proximal joint for respective simulations.

#### Force to counteract *F*_*GRF*_ moment

To calculate the total extensor force *F*_*m*_, (distributed between M. gastrocnemius and M. fibularis longus), moments about the ankle joint were set to 0 so that the ground reaction and extensor moments about the mesotarsal joint were balanced (Fig 17). The center of rotation in lateral view was estimated to be at the midpoints between anatomical and experimental axes determined by Jindrich et al. [11]. Moment arms were measured graphically in OsiriX, with the CT render positioned so that the angles between moment arms and forces were equal to 90 degrees. The vertical component of the extensor force, necessary to counteract the ground reaction moment, was 4202 N. Future analyses will assess contribution of posterior attachments of M. gastrocnemius, which will exert additional moments as long as they are posterior to the transverse axis of joint rotation.

#### Extensor tension *F*_*m*_

The extensor muscle tension *F*_*m*_ was greater than its vertical component, because the resultant of M. gastrocnemius acts along the tibia is angled in 3D relative to the tarsometatarsus. Unknown components and the resultant of *F*_*m*_ were first calculated with the law of cosines [60,61] in the reference frame of the ground, using known vertical component and angles of the tarsometatarsus (Table 5; Figs 15 and 16). The force vector *F*_*m*_ in the global coordinate system was reoriented into the anatomical coordinate system (with the z-axis coincident with the tarsometatarsus long axis) with rotation matrices [27]. For the first rotation about the y-axis, using angles from Table 5 and Fig 18, the matrix (Eq 1) is:

$$R_{y}(a_{x}F_{mx}L1-a_{x}F_{m})=\left[\begin{array}{ccc} cos & 0 & sin\\ 0 & 1 & 0\\ -sin & 0 & cos \end{array}\right]$$ 1)

The second rotation about the x-axis has the matrix (Eq 2):

$${}_{R_{x}(\beta_{y}F_{mx}L1-\beta_{y}F_{m})=\left[\begin{array}{ccc} 1 & 0 & 0\\ 0 & cos & -sin\\ 0 & sin & cos \end{array}\right]}$$ 2)

Table 6 presents components of *F*_*m*_ in the global and anatomical coordinate systems. Table 7 lists muscle forces components applied in all loadcases, plus the ground reaction components.

#### Applying *F*_*m*_ to attachments of the M. gastrocnemius tendon

**Fig 20:**
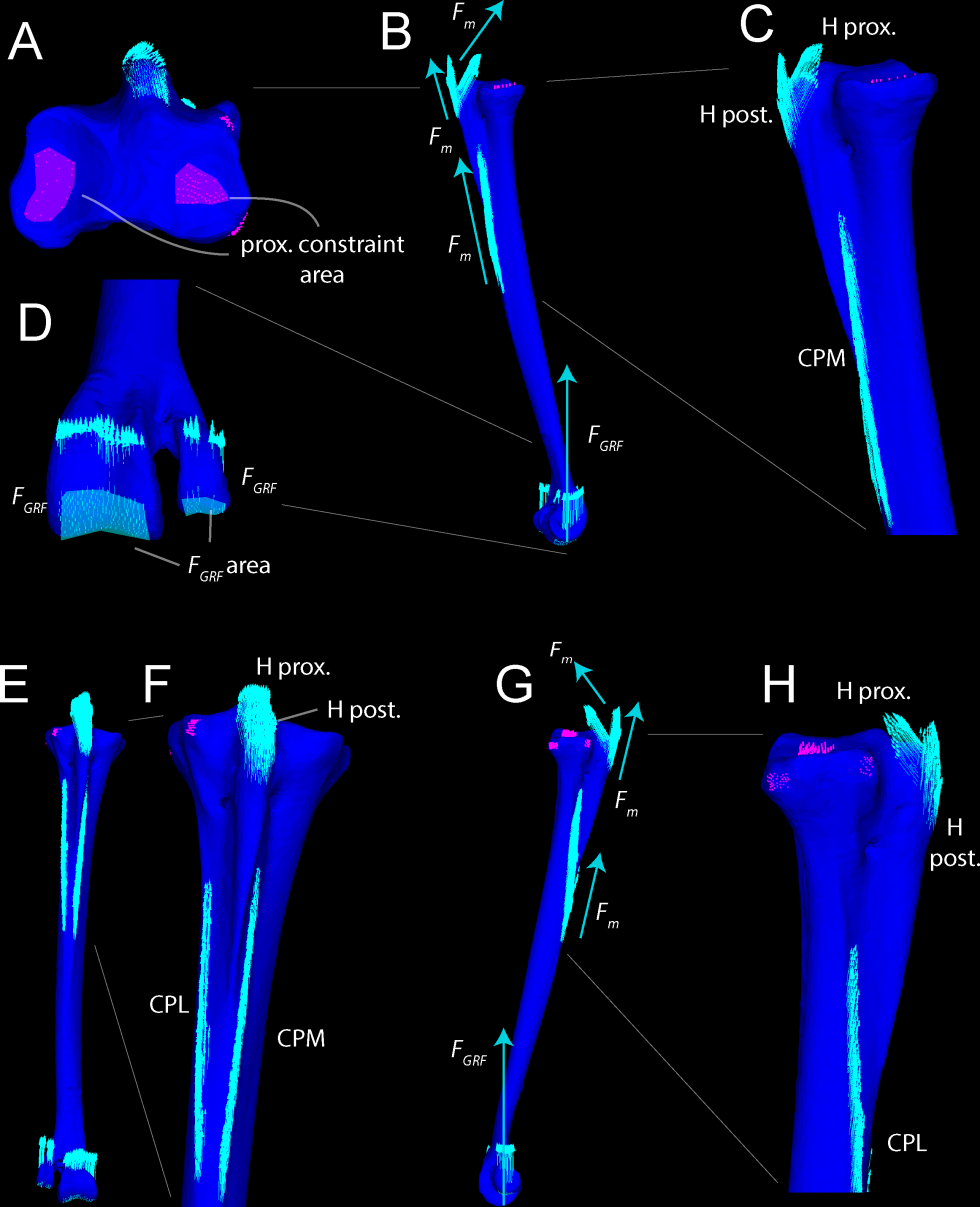
Constraints (pink dots and regions) and resultants of forces applied to nodes (teal arrows) for FEA are shown in proximal (A), medial (B and C), anterodistal (D), posterior (E and F), and lateral (G and H) views. In D, the large teal area shows the tails of the arrows where the *F*_*GRF*_ was applied. Large arrows in B and G represent tension *F*_*m*_ in the gastrocnemius tendon, and the ground reaction force *F*_*GRF*_. H prox. = proximal surface of the hypotarsus. H post. = distal surface of the hypotarsus, CPL = Crista plantares lateralis, CPM = Crista plantares medialis.

Fig 20 diagrams all forces and constraints applied to the FE model, including distribution of *F*_*m*_. The magnitude of *F*_*m*_ was assumed to be constant in the M. gastrocnemius tendon, and intuitively would be divided equally among all nodes of its attachments to the hypotarsus and the posterior surface of the tarsometatarsus. Directional components of *F*_*m*_ on the hypotarsus were calculated as described above (Figs 15 and 16) but components on the Crista plantares lateralis and Crista plantares medials (not in line with the direction of pull on the hypotarsus) were not obvious. To simulate displacements of the tendon along its attachments to the posterior surface (Fig 3), we applied masking tape to the attachments on a *Struthio* tarsometatarsus (Ohio University Vertebrate Collections OUVC 4023), with a free end replicating the tendon as it approaches the hypotarsus. We marked the bone and masking tape with pencil, positioned the metatarsus at the proper angles, and pulled on the free “tendon” in the resultant direction of *F*_*m*_. Markings at the proximal portion of the hypotarsus became displaced in the direction of pull. Markings at attachments more distally on the hypotarsus, and to the posterior cristae, were displaced almost exclusively along the bone’s long axis, with negligible lateral displacement. At the posterior nodes of M. gastrocnemius attachment, we therefore restricted tension of M. gastrocnemius to the z, long axis of the bone.

## Appendix 1: Stress and strain theory and notation

Stress, the internal force per unit area of a material, in a three dimensional structure is accounted for with a stress tensor, which records stresses in three directions (*x*, *y*, *z*) on three orthogonal surfaces (*x*, *y*, *z*). These stresses are conventionally and conveniently represented in matrix form, in Eq 3.

$$\sigma =\left(\begin{array}{ccc} \sigma_{xx} & \sigma_{xy} & \sigma_{xz}\\ & \sigma_{yy} & \sigma_{yz}\\ sym & & \sigma_{zz} \end{array}\right)$$ 3)

Normal stresses (on the diagonal of the matrix), *σ*_*xx*_, *σ*_*yy*_, *σ*_*zz*_, occur where compressive (crushing) or tensile (pulling) stress is perpendicular to a surface in the x, y, or z directions, and thus parallel with these axes. Shear stresses *σ*_*xy*_, *σ*_*xz*_, *σ*_*yz*_, are parallel to a surface but perpendicular to each other. For example, *σ*_*xy*_ signifies that in the xy plane, stress is in the x direction relative to the y axis. This shear is analogous to having children’s toy blocks stacked vertically (on the y axis), and pushing on a block in the x direction (perpendicular to the y axis). Because a shear stress such as *σ*_*xy*_ is equivalent to *σ*_*yx*_, the stress tensor is symmetric and designated by *sym* in the matrix notation.

von Mises theory is a common choice for reducing the stress tensor to a single value that can be compared to experimental results of a uniaxial tensile test, and reflects the likelihood of material failure. This theory recognizes that many materials fracture not due to stresses that change the volume (hydrostatic or volumetric stresses), but rather those that change the shape (distortional stresses). The von Mises equivalent stress is a scalar value that reflects these distortional stresses (Eq 4).

$$\sigma_{vM}=\sqrt{0.5[{\left(\sigma_{xx}-\sigma_{yy}\right)}^{2}+{\left(\sigma_{yy}-\sigma_{zz}\right)}^{2}+{\left(\sigma_{zz}-\sigma_{xx}\right)}^{2}+6(\sigma_{xy}^{2}+\sigma_{yz}^{2}+\sigma_{xz}^{2})]}$$ 4)

Strain is a measure of relative deformation (change in length divided by original length), which also takes a form of a tensor of 6 unique values, (*ε*_*xx*_, *ε*_*yy*_, *ε*_*zz*_, *ε*_*xy*_, *ε*_*xz*_, *ε*_*yz*_). Von Mises strain can be calculated with the same equation above, substituting strain components for stress.

The levels of von Mises stress *σ*_*vm*_ and strain *ε*_*vm*_ relative to a material’s yield values *σ*_*yield*_ and *ε*_*yield*_ indicate how close a structure is to nonlinear deformation and therefore damage, and their approach to ultimate values *σ*_*ult*_ and *ε*_*ult*_ indicate how close the structure is to immediate fracture. Dumont et al. [24] and Rayfield and Milner [25] cogently discuss von Mises criteria as they relate to bone. We assessed the mechanical integrity of the ostrich tarsometatarsus by comparing simulated stresses and strains to *σ*_*ult*_ and *ε*_*ult*_ of compact and cancellous bone. *σ*_*ult*_ and *ε*_*ult*_ are unknown for bone of the ostrich tarsometatarsus, and were assumed to be similar to those of emu, human, and bovine cortical bone [55,36,40], and human and dog appendicular cancellous bone [62,63,64,28].

## Supporting Information

S1 Dataset Strand7 finite element model of *Struthio* tarsometatarsus, with compact and cancellous bone properties. This FE mesh has 77,722 nodes and 386,953 tetrahedra. It is the maximum size for PLOS ONE supplementary files. Higher-resolution models are available from the second author. This model gives realistic strain results within cancellous and compact bone, but unreliable results where the materials meet.
(ST7)

S2 Dataset Strand7 finite element model of *Struthio* tarsometatarsus, with only compact bone properties. This FE mesh has 77,722 nodes and 386,953 tetrahedra. It is the maximum size for PLOS ONE supplementary files. Higher-resolution models are available from the second author. This model’s uniform properties give realistic stress distribution, but misleading strain results at the proximal and distal ends of the element.
(ST7)

S3 Dataset Surface model of the *Struthio* tarsometatarsus, for solid meshing in FEA programs. The tetrahedra can be smoothed in programs such as Mimics, Avizo, and Geomagic.
(STL)

S4 Dataset CT scan of *Struthio camelus* tarsometatarsus. The dataset “UCMZ_Struthio_adult_tarsometatarsus-DICOM_CT.zip” contains DICOM CT data from the *Struthio* tarsometatarsus scan.
(ZIP)
